# Wealth and Child Mortality in the Nineteenth-Century United States: Evidence from Three Panels of American Couples, 1850–1880

**DOI:** 10.1017/ssh.2023.12

**Published:** 2023-06-23

**Authors:** J. David Hacker, Martin Dribe, Jonas Helgertz

**Affiliations:** 1 Institute for Social Research and Data Innovation, University of Minnesota, Minneapolis, MN, USA; 2 Department of Economic History and Centre for Economic Demography, Lund University, Lund, Sweden

## Abstract

With only a few exceptions, the historical study of individual-level correlates of child mortality in the United States has been limited to the period surrounding the turn of the twentieth century, when children ever born and children surviving data collected by the 1900 and 1910 censuses allow indirect estimation of child mortality. The recent release of linked census data, such as the IPUMS MLP datasets, allows a different type of indirect estimation over a longer period. By following couples across subsequent decennial censuses, it is possible to infer child mortality by measuring whether couples’ own children in the first census were still present in the second census. We focus our analysis on children aged 1–3 in the first of two linked censuses, who were less likely to be undercounted by the census than infants, and unlikely to be living apart from their parents in the second census. We estimate child mortality over the intervening decade and use OLS regression to correlate that mortality to the residence location and socioeconomic characteristics of their parents’ households. We limit our analysis to three panel datasets for married couples linked between the 1850–60, 1860–70, and 1870–80 censuses, when real estate and personal estate wealth data were collected. Our results indicate a significant negative relationship between wealth and child mortality across all regions of the United States and over the entire period examined.

## Introduction

All over the Western world today, socioeconomic status is positively associated with health and negatively associated with mortality (e.g., Mackenbach et al. 1997; Cutler et al. 2012; Chetty et al. 2016; Mackenbach 2019). The historical origin of this “health gradient” is less certain. Some researchers have argued that socioeconomic differentials in mortality were as big, or even bigger, in the past as they are today (Antonovsky 1967; Marmot 2004; Deaton 2016). In several influential articles, Link and Phelan (1995, 1996) argued that social conditions are *fundamental cause*s of disease and mortality, persisting over time despite changes in disease environments, risks, and the mechanisms linking socioeconomic status and mortality (see also Clouston et al. 2016). Other researchers, however, have claimed that socioeconomic differences in mortality were small historically because the diseases responsible for much of the mortality were highly communicable (Smith 1983; Livi-Bacci 1991).

For childhood mortality, the evidence on socioeconomic differences in the past is mixed and differs by context. Jaadla et al. (2020), for example, reported a significant but non-linear relationship between child mortality and an occupation-based measure of parents’ economic well-being in mid-nineteenth century England, while Dribe and Karlsson’s recent investigation of long-term trends in child mortality in Sweden (2022) found no evidence for a mortality gradient by socioeconomic status (also based on occupation) until after the onset of the mortality transition in the late nineteenth century. Although children born to parents living in extreme poverty clearly suffered a higher risk of death than other children in all contexts, there is no evidence for a socioeconomic gradient in childhood mortality in Western societies before the mortality transition, if by gradient we mean a consistent negative association between status and mortality across the whole status distribution and not just a difference between the lowest status groups and the rest. As in other areas of historical demography, however, research is limited by the quality and quantity of available data. High-quality data on parents’ economic well-being is especially lacking. More studies are needed, particularly more research on the period prior to the onset of the mortality transition.

In this study, we examine the relationship between parental wealth and child mortality in the mid-nineteenth-century United States immediately prior to the onset of sustained mortality decline, which began about 1880 (Haines 2020). We rely on new IPUMS Multigenerational Longitudinal Project (IPUMS MLP) data (Helgertz et al. 2020, 2022) to construct panel datasets for over 2 million married couples linked between the 1850–60, 1860–70, and 1870–80 censuses. The panel datasets allow us to estimate child mortality using the observed survival of couples’ children over the intercensal intervals. In addition to describing a new approach of indirectly estimating child mortality using linked censuses, a major contribution of this study is its inclusion and analysis of wealth data, which were collected for all individuals in the 1850, 1860, and 1870 censuses. Wealth is a more direct measure of economic well-being than father’s occupation, which has been used frequently in historical studies as a proxy of parents’ income, wealth, class, and social status (e.g., Garrett et al. 2001; Dribe et al. 2020; Jaadla et al. 2020). We look at parents’ real estate wealth, personal estate wealth, and combined wealth and study the association between wealth decile and child mortality, controlling for confounding factors. In addition to analysis at the national level, we make separate analyses by census region. Compared to previous studies, we use national data and a substantially larger number of cases, which allows a more detailed analysis of the association between wealth and mortality in different parts of the nation.

## Background

Although a negative relationship between socioeconomic status and mortality has been consistently observed in modern societies (Marmot 2004; Elo 2009; Deaton 2016), the precise causes of these associations have been disputed (House et al. 1988; Marmot et al. 1991; Smith 1999; Kuh and Ben-Shlomo 2004). Some researchers have contended that social conditions are fundamental causes of disease and mortality wherever access to resources is unequal and diseases can be avoided (Link and Phelan 1995, 1996; see also Clouston et al. 2016). Although disease environments, causes of death, and pathways connecting socioeconomic factors and mortality have varied over time, according to this perspective, socioeconomic conditions have remained fundamental causes, resulting in persistent inequalities in health and mortality.

Historical research on the relationship between socioeconomic status and child mortality, however, has failed to find a strong and consistent relationship between parents’ socioeconomic status and child mortality prior to the late nineteenth century, casting some doubt on the long-term applicability of fundamental cause theory. Steckel (1988) and Ferrie (2003), for example, found no consistent association between socioeconomic status and childhood mortality in the mid-nineteenth-century United States, whether measured by occupation, wealth, or literacy. The empirical basis for drawing firm conclusions was thin, however, being based on small and regional samples of census and retrospective mortality data. In a recent analysis of child mortality in southern Sweden, Dribe and Karlsson (2022) found no social class differences in infant and child mortality in the early nineteenth century. Class differentials emerged only in the late nineteenth century in the context of industrialization, economic development, and mortality decline. A few studies have reported mixed evidence in support of a mortality gradient. In their study of child mortality in a mid-nineteenth-century parish in Tuscany, Italy, Breschi et al. (2004) found a weak socioeconomic gradient in infant mortality and a stronger gradient in the mortality of older children. Similar socioeconomic differences in child mortality have also been documented in early-nineteenth century England. Results, however, were nonlinear and explained more by the disease environment than by socioeconomic status (Jaadla et al. 2020; Razzel and Spence 2006).

Preston and Haines provided the earliest evidence of a negative association between socioeconomic status and child mortality in the United States in *Fatal Years: Childhood Mortality in Late Nineteenth-Century America* (1991) using a public-use microdata sample of the 1900 U.S. census. The census collected information on the number of women’s children ever born and the number of children surviving, which allowed Preston and Haines to estimate and construct empirical models of child mortality at the level of individual mothers with controls for individual-level, household-level, and area-level correlates. The results showed only small and inconsistent differentials in child mortality by socioeconomic status, as measured by the father’s occupation, home ownership, and unemployment. The difference in mortality between children of white-collar and blue-collar workers was not large, and most of the differences between occupational groups were related to place of residence, nativity, or race (e.g., low mortality for children of farmers and high mortality for children of black urban laborers in the South). Preston and Haines attributed the relatively small socioeconomic differences to the poor knowledge about disease transmission in society and medical care, which made it difficult for high-status groups to protect their children from disease and death.

Similar census data documenting the number of children ever born and children surviving were collected in the 1910 census of the United States and the 1911 censuses for Ireland and England and Wales, which has supported similar analyses with similar results. Preston et al.’s analysis of the 1910 public-use sample (1994), which was constructed with a higher density sample than the 1900 sample, found large mortality differences across ethnic groups (e.g., low mortality among children of Jewish parents and high mortality among children of French-Canadian parents) and relatively small differences across occupation groups. In England and Wales, as well as in Dublin, Ireland, childhood mortality was clearly associated with social class (based on occupation), but the contexts in which people lived were often more important than class and confounded the class-mortality association (e.g., Preston and Haines 1991; Woods et al. 1993; Haines 1995; Woods 2000; Garrett et al. 2001; Cormac 2004; Connor 2017). There was no trend over time in mortality differentials in the Netherlands, but poorer regions had more pronounced class differences than richer regions (Van Poppel et al. 2005).

A stronger and more consistent negative association between socioeconomic status and child mortality emerged in the later stages of the mortality transition. Woodbury’s classic study of infant mortality in the United States (1926), for example, identified a large and consistent mortality gradient across income groups. Among infants born to native-born white mothers, and whose fathers’ earnings exceeded $1,250 annually, the mortality rate was 58 per thousand live births; among infants whose father’s earnings were less than $450, the rate was 170 per 1,000 live births. In Sweden, the socioeconomic differentials that emerged in the late nineteenth century also developed into a full gradient in childhood mortality in the twentieth century (Dribe and Karlsson 2022).

From a theoretical perspective, socioeconomic status can affect the health and mortality of children through several pathways, related to maternal factors (birth interval and birth order), nutrition, health care, injuries, and environment (e.g., Mosley and Chen 1984). Economic resources improve nutrition and access to health care and reduce exposure to infectious diseases through more hygienic living quarters and access to clean water and sanitation (see, e.g., Edvinsson 2004). Socioeconomic status may also interact with cultural factors in determining different behavior with consequences for child health, such as fertility, breastfeeding, uptake of health innovations, food preparation, and lifestyles. However, many of these variables are also confounded by contextual settings, such as publicly provided services like water and sanitation facilities, and health care access, as well as food security (Antonovsky and Bernstein 1977; Sundin 1995; Preston et al. 1998).

As mortality started to decline, there was also a change in disease patterns, often described as “the epidemiological transition” (Omran 1971). Before the transition, infectious diseases transmitted by air, food, or water dominated among the causes of death. People’s knowledge about what caused disease was rudimentary and sometimes even inaccurate (Preston and Haines 1991; Easterlin 1999), which meant that individuals who otherwise might have had the resources to protect themselves or their children from diseases and death, such as the higher-status groups, were unable to do so (Smith 1983). In the late nineteenth-century United States, for example, children of physicians experienced only 6% lower mortality rates than children of other parents, while the children of teachers had no advantage at all (Condran and Preston 1994). In the first stages of the mortality transition, smallpox vaccination (in the early nineteenth century) and improved sanitation in the cities (from the mid-nineteenth century) reduced deaths from smallpox and various waterborne diseases, respectively (Sköld 1996; Troesken 2004). As knowledge of disease transmission improved, most notably through the acceptance of the germ theory of disease from the 1880s (Preston and Haines 1991; Easterlin 1999), mortality from infectious diseases was further reduced, and then continued to decline thanks to the medical innovations in the twentieth century (see Omran 1971).

The fact that infant and child mortality was high even in high-status groups, where diet and nutrition must have been satisfactory, has led to a questioning of the idea that the mortality decline was caused by improved nutrition (McKeown 1976). Instead, much of the focus in the literature has been on the role of disease environment, maternal care (especially breastfeeding), water and sanitation, and early public health initiatives as leading explanations of the declines in childhood mortality that occurred before the real breakthrough of modern medicine (e.g., Szreter 1988; Preston and Haines 1991; Reid 1997; Woods 2000; Razzel and Spence 2006). As new health interventions became available and knowledge improved, higher-status groups could be expected to have benefitted first, leading to a sharpened mortality gradient (Morris and Heady 1955; Antonovsky and Bernstein 1977; Cutler et al. 2012) or even to the emergence of such a gradient in the first place (Smith 1983). As health knowledge diffused to lower-status groups, urban infrastructure improved, and new health interventions became more accessible and affordable, it could perhaps be expected that the importance of socioeconomic status declined. For example, adequate provision of clean water and sanitary facilities for the whole population, through public provisions, would reduce socioeconomic disparities. Both black and white children benefitted from improved water supplies and sewage in the early twentieth century South, leading to a narrowing of the race differential in child mortality (Troesken 2002, 2004). Similarly, the establishment and expansion of publicly funded universal health care would reduce the gradient, since access was less dependent on economic status.

The period we study, however, predates many of these developments and the onset of significant mortality decline, which Haines (2000) has identified as beginning in the United States during the 1870s, the period covered by the last of our three panel datasets. In this context, wealthier parents would have found it difficult to leverage their resources to shield their children from communicable diseases or to treat acquired illnesses. To cite just one example from the period, Abraham Lincoln’s second son Eddie died of “chronic consumption” (likely tuberculosis) in 1850 at age three, despite Lincoln’s considerable wealth earned as a lawyer, and his third son Willie contracted and died in 1862 from “bilious fever” (likely typhoid fever) while living in the White House, despite the care of three of Washington, D.C.’s best physicians (Dirck 2019).

Our study, however, does not predate the beginnings of the sanitation movement in the United States (Duffy 1992), a growing emphasis in the period 1750–1900 on cleanliness and domestic hygiene to promote health (Bushman and Bushman 1988; Tomes 1990), or the early phases of the fertility transition, which began among “Yankee” couples living in New England circa 1840 (Hacker 2016). Although early sanitary reforms were limited by lack of knowledge of germ theory and likely benefited all classes, the greater emphasis on cleanliness among upper- and middle-class parents could have contributed to higher survival rates among their children. Recent analyses of linked census data have indicated an inverse “U-shaped” relationship between wealth and marital fertility in the period 1850–80 (Hacker et al. 2021), suggesting that wealthier couples were consciously limiting their fertility, which also could have had indirect benefits on the health and survival of their children.

To summarize, there does not seem to have been a consistent association between socioeconomic status and childhood mortality in Western societies before, or early in, the mortality transition. When the transition was well underway, which also coincided with industrialization in many places, socioeconomic differentials emerged, as the higher-status groups experienced an earlier, or sometimes faster mortality decline. Gradually these differentials developed into a full health gradient. Based on this evidence we should not expect childhood mortality in the mid-nineteenth century United States to have varied markedly by wealth or other measures of socioeconomic status. It is possible, however, that the growing emphasis on cleanliness and domestic hygiene among middle-class and upper-class parents and the early fertility transition with its clear socioeconomic pattern had implications for mortality differentials because of the association between fertility and child survival.

## Data

We relied on three panel datasets of married couples constructed using IPUMS Multigenerational Longitudinal Project (MLP) data covering the periods 1850–60, 1860–70, and 1870–80 (Helgertz et al. 2020). The IPUMS MLP datasets and their methods of construction have been described in detail elsewhere (Helgertz et al. 2022). Briefly, the datasets consist of census “crosswalks” identifying individuals linked between two or more of the IPUMS full-count census datasets (Ruggles et al. 2021). Individuals were linked using machine-learning algorithms that relied on time-invariant information captured in both censuses (individuals’ first name, last name, race, sex, and place of birth), supplemented with information related to the local context and other household members to improve accuracy. An evaluation of the accuracy of the method used to generate the IPUMS MLP links indicates a considerably lower type I error rate than other methods of automated record linkage used on U.S. census data (Helgertz et al. 2022), a dramatically lower rate of false positives than incurred by other projects employing similar automatic linking methods (e.g., Abramitzky et al. 2021).

From each of the three panel datasets we selected an initial analytical population limited to married couples with: (1) spouses successfully linked between the two censuses; (2) spouses who remained married to each other in both censuses; and (3) one or more own children aged 0–9 in the first of the two censuses, who we considered to be “at risk” of mortality in the intercensal period. Using this dataset, we conducted several preliminary analyses of our ability to estimate the mortality of the at-risk children from their observed survival to the second census, when aged 10–19. Ultimately, as discussed in more detail below and in the Online Appendix, we concluded that our indirect estimation method worked best for children aged 1–3 in the first census, who were well enumerated in both censuses (children aged 0, in contrast, appeared to have been frequently undercounted by the first census) and who were more likely, assuming they survived the decade, to be living in their parents’ households in the second census than their older surviving siblings, thereby increasing the probability they would be linked by the IPUMS MLP project and known to survive. Our final analytical dataset, therefore, consisted of linked married couples with one or more children aged 1–3 in the first census, who were the only children considered at risk of death in the subsequent decade. Because approximately 9-in-10 Black individuals living in the United States in 1850 and 1860 were enumerated on a separate schedule for enslaved inhabitants without names, we limited the analysis to white couples.

By requiring that both spouses were linked and survived each decade, we reduced the already low chances of type 1 errors in the linked datasets and increased the chances that surviving children, relative to children born to parents whose marriages were disrupted by the death of one or both partners, would be living in their parents’ households and linked by the IPUMS MLP project. Although we did not explicitly require couples’ surviving children to be linked between the two censuses, a large majority of children present in the second census who were old enough to have been enumerated in the first census were linked as well.

Linked census records are unlikely to be representative of the populations from which they are drawn, even if methods used to establish the links relied solely on time-invariant matching criteria (Antonie et al. 2020). Unfortunately, our requirement that couples survived each decade makes it impossible to make direct comparisons between couples in the linked analytical datasets and couples in the overall population using the same selection criteria. We have no means of determining – other than from the IPUMS MLP dataset itself – which couples in the overall population survived each decade. In addition, our selection criteria implicitly assumes that couples were still living in the United States 10 years after the first census, when they were at risk of being enumerated by the next census, and we have no means of determining which couples remained resident in the country. Consequently, Table 1, which compares couples in the three linked MLP datasets with similar couples in the corresponding IPUMS cross-sectional datasets, is not a comparison of the linked and overall targeted populations, but a comparison of the linked datasets and proxies of the overall targeted populations. An unknown but likely significant number of the couples in the cross-sectional datasets were in marriages that would soon be disrupted by death, divorce, or separation, and an unknown number of couples would be living outside the United States at the time of the next census (a significant problem when analyzing foreign-born couples, who were at high risk of returning to their country of origin and therefore more likely to be unobserved in the second census than U.S.-born couples). These couples likely differed in significant ways from couples in the targeted populations of surviving couples, biasing comparisons.

Given these caveats, the results indicate that couples in the panel datasets closely resembled couples in the cross-sectional datasets, with some modest differences in expected areas. Couples in the linked panel datasets were moderately more likely to be literate, born in the United States, living in rural areas, living on a farm, living in the Northeast census region, and had more children living in the household (although the number of children under age 5 was very similar) than couples in the cross-sectional datasets. Linked couples also had approximately 15% greater wealth than couples in the cross-sectional datasets. Overall, however, the differences were modest and might have resulted from unobserved differences in couples’ survival over the census interval, not biases incurred in the linking process. Rural couples and wealthier couples in the cross-sectional dataset, for example, might have been more likely to survive the decade than urban couples and poorer couples. We would therefore expect to link a higher percentage of these couples to the second census, even if the automatic linking algorithms linked the same percentage of surviving couples. We therefore did not weight our analytical datasets to represent the cross-sectional populations as suggested by some researchers (Antonie et al. 2020; Bailey et al. 2020). Although it was theoretically possible to do so, weighting the dataset had the potential to make our results *less* representative of the targeted population of surviving couples who remained in the United States. Nevertheless, we caution that the results presented below are strictly applicable only to the population of linked couples. In the Online Appendix we show our results for the 1870–80 panel dataset using weights derived from inverse propensity scores (Bailey et al. 2020) to yield statistics representative of the cross-sectional population in 1870. These results correspond very closely to the results presented below and had no effect on our conclusions. In the specific case of the relationship between wealth and child mortality the results were nearly identical.

At the bottom of Table 1, we make rough estimates of the overall size of the potentially “linkable” population, defined as couples who survived the intercensal interval and were enumerated by both censuses. To estimate the size of the linkable population we relied on published ten-year survival estimates and census undercount estimates for the native-born white population (Hacker 2010, 2013). Because our data includes foreign-born couples – who likely suffered higher mortality rates and were more likely to be missed by the subsequent census – our estimates of the size of the linkable population were likely biased upwards (and the corresponding linkage rates downwards). The results indicate that our linking methods identified between 62% and 69% of the potentially linkable population, depending on the census interval. The true linkage percentage was likely higher. Among the children enumerated in the second of the two censuses who were old enough to be enumerated in the first census, 84% were linked to the first census. Unlinked couples and unlinked children either had several potential matches (e.g., two or more potential matches for couples with common names), were enumerated poorly (e.g., with an inaccurate age, misspelled name, inaccurate birthplace, or only an initial), were transcribed poorly, or (in the case of the unlinked children) not enumerated in the first of the two censuses.

### Measuring child mortality

Our basic approach is easily described. To estimate child mortality, we began by restricting the analytical datasets to married couples linked between the 1850–60, 1860–70, and 1870–80 censuses who had one or more coresident own children in the first of the two linked censuses (hereafter, Census “A”). We then determined the number of those children who survived to the second of the two censuses (hereafter, Census “B”) using the links provided by the IPUMS MLP project. Finally, we assumed that children in Census A who were *not* linked to Census B died in the ten-year interval between the two censuses. Dividing the number of children dying by the number of at-risk children in Census A resulted in the proportion of each couple’s children dying in the ten-year interval.

This basic approach is subject to several sources of error, which we minimized by restricting our analysis to a narrow age group of at-risk children and by making a few corrective adjustments to the data. An initial exploratory analysis indicated several potentially important sources of error: (1) failures by the IPUMS MLP project to link a few children present in both censuses; (2) differences in the census under-enumeration of children, especially among children under the age of 1; and (3) unobserved differences in the age at which children left their parents’ home. This initial analysis is described in the Online Appendix.

Briefly, our analysis indicated that the IPUMS MLP, while taking steps to limit type I errors, was too conservative in linking children of married couples who could be safely assumed to be the same child. We therefore forced links among couples’ unlinked children with the same sex and approximate birth years in both censuses. In 1870, for example, Missouri couple Joab and Elizabeth Hobson had three male children, named “A L,” “A J,” and “F S,” aged 4, 4, and 1, who were not linked forward to a child in the 1880 census. In 1880, Joab and Elizabeth had three male children, named “Abraham,” “Andrew,” and “Phillip,” aged 14, 14, and 11, all born in Missouri, who were not linked backward to a child in the 1870 census. We forced links between these children and similar unlinked children with the same sex and approximate birth years of other linked couples. We then used children aged 10–19 in Census B to estimate census undercounts among children aged 0–9 in Census A, under the assumption that children coresiding with their mothers and fathers when aged 10–19 also should have been present in their parents’ households a decade earlier when aged 0–9. These estimates indicated that children under the age of 1 were much more likely to be missed by the 1850, 1860, and 1870 censuses than children aged 1–9. Although we were able to estimate and adjust for each couple’s undercounted children, the adjustments were large for children aged 0, and we had no alternate means of evaluating their accuracy. We therefore decided to drop children aged 0 in Census A from the analysis.

Finally, our preliminary analysis also indicated that children who were still living in Census B but who had left their parents’ homes were less likely to be linked between the two censuses by the IPUMS MLP project. If not linked, these children will appear in our analysis to be deceased. Because the propensity for children to leave their parents’ households likely varied by the wealth and other characteristics of their parents, the departure of children from their parents’ homes could bias our results. Unfortunately, the age a couple’s children left home was unobserved and can only be estimated at the population level using model life tables and an assumed level of mortality. Steckel (1996) has estimated that the median age of leaving home in the period 1850–60 was 26 years for white males and 25 years for white females, but also concluded that a small percentage of boys and girls left home before aged 16. Although we had no means of estimating how differences in the age of leaving home varied by wealth, our exploratory analysis indicated that age-specific 10-year survival probabilities of children closely corresponded with model life table for children aged 3 and younger in Census A (aged 13 and younger in Census B), but then increasingly diverged from the expected age pattern of survival for children aged 4 and above in Census A (aged 14 and above in Census B). Although the divergence was small for children aged 4 in Census A, we decided to drop children aged 4 and older from the analysis. Together with our concern about the relatively large undercount of children aged 0 in Census A, our final analytical population was limited to linked couples with children aged 1–3 in Census A. Although this narrow age group of at-risk children limits the number of cases available for analysis, the large size of the MLP datasets ensures that we have large numbers of cases for analysis.

A more detailed discussion about possible biases and alternative measurements of child mortality, including the sensitivity of the results to the reliance on different age groups of children, is provided in the Online Appendix and concludes that our findings are robust to alternative assumptions.

### Measuring parental wealth

Most historical studies of child mortality have relied on fathers’ occupation or occupational group as a measure of socioeconomic status, or economic well-being more generally (e.g., Dribe et al. 2020; Garrett et al. 2001). Although occupations were correlated with income and have been used to assign income scores to individuals in historical censuses (e.g., Sobek 1995), there were likely unknown but significant heterogeneities within occupations in terms of income and wealth. This represents a serious limitation in the mid-nineteenth-century United States, when approximately half of all fathers were enumerated in the census as “farmers.” Wealth data recorded by the 1850, 1860, and 1870 censuses allow us to examine the relationship between parents’ economic well-being and child mortality more directly. It is likely, of course, that economic status was also correlated to some extent with social status, and hence that wealth also captures other aspects of social position than economic living standards.

We first combined wives’ and husbands’ real estate, personal estate, and their total combined wealth, if reported separately, into a measure of couples’ real, personal, and total wealth, measured in United States dollars unadjusted for inflation, which we then divided into deciles for each census year to the extent possible. Although women’s property acts were enacted by an increasing number of states following the passage of the first such act in Mississippi in 1839, nineteenth-century coverture laws typically meant that all property held by women prior to their marriage became the property of their husbands at marriage. Reporting practices by the enumerators also clearly assumed all wealth held together by a couple was reported on the husband’s record. In each census, less than 1% of married women had wealth reported separately. Because wealth typically increased with the husband’s and wife’s age, we included women’s age and spouses’ age differentials in regression models.

Enumerator instructions stated that only real and personal estate wealth above $100 were to be recorded, so each wealth variable is effectively truncated below that value. On average, nearly half of all households in 1850 with a childbearing woman reported no real estate wealth, although the proportion varied by subgroups (e.g., “farm couples,” which included a husband whose occupation was recorded as a “farmer” or “farm laborer,” were more likely to own real estate). Depending on the census year and wealth variable, the percentage of couples reporting no wealth in national models ranged from a high of 44%, in the case of couples’ real estate wealth in the 1870–80 dataset, to a low of 12%, in the case of couples’ total wealth in the 1860–70 dataset.

Nineteenth-century census officials lacked the resources to analyze the wealth results and some officials, including Census Superintendent Francis Amasa Walker, expressed skepticism that wealth was accurately reported or that the questions were worth the resources spent for their collection (Magnuson 1995: 58–9). Census enumerators were told to ignore mortgages and liens. Although mortgages were less common in the nineteenth century – Snowden (2006) estimates about 30% of properties in the period were mortgaged – there is some possibility that wealth estimates are biased. In addition, although the censuses were never used to assess direct taxes, returns were not private, and some individuals may have hesitated to report their true wealth, especially their true personal estate wealth, which was easier to conceal and less evident to observers. Distrust in the census among nineteenth-century individuals was generally low, however. Most respondents probably tried to give an accurate approximation. Richard Steckel’s (1994) comparison of wealth reported by individuals in the 1870 census with independent assessments made by assessors in Boston, Salem, Lexington, Westminster, and Sturbridge, Massachusetts, indicated similar wealth distributions and high correlations between the two measures.

Before conducting our analysis, we made some preliminary examinations of the wealth data. On aggregate, the data appear reasonably accurate. Nationally, mean wealth rose steadily with individuals’ age until about age 45–50, rose at a slower pace to about age 65, after which it began to decline. This age pattern is expected; men’s age-related debilities increased after middle age, work hours and incomes declined, and wealth bequests were increasingly made to children reaching adulthood (Kearl and Pope 1983). Although occupational income scores for each occupation (the mean annual income for men reporting each occupation) are not available in the census until 1950 (Sobek 1995), application of those income scores to the nineteenth-century occupations (using the IPUMS “occscore” variable) indicated that wealth and occupation income scores were strongly correlated, increasing confidence in the wealth data (see the Online Appendix for more information on the age-pattern of wealth and the correlation between occupational income scores and wealth). In addition, wealth differentials by income, nativity, and region were consistent with the economic and social history literature, with greater wealth among native-born couples relative to foreign-born couples and greater wealth in the South in 1860 relative to other regions, where the rapidly growing slave population increased slaveowners’ personal estate wealth and where market returns from cotton were high. As expected, the American Civil War and the emancipation of slaves in the war’s aftermath took a major toll on white men’s wealth in the South, with dramatically lower wealth in the 1870 census relative to the 1860 census (Dupont and Rosenbloom 2022). Our interpretation is that while wealth might be inconsistently recorded for individuals, on aggregate the results should be interpretable. Any measurement error at the individual level will tend to deflate the significance of wealth variables in empirical models and increase the chances of accepting the null hypothesis. Our results, therefore, will tend to be conservative.

### Descriptive statistics

Table 2 shows the mean proportion of children aged 1–3 dying by father’s occupation group, region, rural–urban residence, and parents’ literacy, nativity, and total combined wealth tercile in the three panel datasets. Overall, the results indicate that child mortality was lowest in the 1870s, which coincides with the approximate timing of the onset of the mortality transition (Haines 2000). Mortality was higher in the 1860s than in the 1850s. The increase in mortality between the 1850s and 1860s may have been a consequence of the American Civil War (1861–65), which is known to have facilitated the spread of infectious diseases. The 1866 cholera epidemic may have also contributed to higher child mortality rates in the 1860s (Rosenberg 1987). About 9% of children aged 1–3 in 1850 and 1870 died prior to the subsequent census. This corresponds to Model West level 9 in the 1850s and Model West level 10 in the 1870s, and are close to mortality levels estimated elsewhere (Hacker 2010). The 11% dying in the 1860s roughly corresponds to Model West level 7, a higher level of mortality than is typically estimated for the period.

Within categories, mortality was lower among children whose fathers were farmers, whose parents were in the upper tercile of total wealth, and who lived in a rural area and the Midwest census region. Mortality was higher among children whose fathers were service workers and general laborers, among children whose parents were in the lower tercile of total wealth, and among children who lived in larger urban cities and in the Northeast census region. The results show little differences among children born to literate and illiterate parents and, in contrast to research for the turn of twentieth century (Preston et al. 1994; Dribe et al. 2020), relatively little advantage among children born to native-born parents relative to foreign-born parents. Many of these variables were intercorrelated, of course. Literate parents, for example, were more likely to live in urban areas. In a study of aggregate mortality in the period 1830–60, Haines et al. (2003) found higher crude death rates in counties with higher per capita wealth, which were also counties with greater urbanization and transportation links. To assess the importance of parents’ wealth independent of other covariates, we turn to regression analysis.

## Regression analysis

We model the proportion of children dying between Census A and Census B as a function of each couple’s socioeconomic characteristics in Census A – including their real estate wealth, personal estate wealth, and combined total estate wealth – to examine the possible relationship between wealth and child mortality. We use weighted OLS regression to model child mortality in each dataset. We weight the results by the number of children aged 1–3 in Census A at risk of death, a standard approach in the literature that reduces the problem of heteroskedasticity and gives more weight to couples with more children, just as they do in overall levels of child mortality in the population (Preston and Haines 1991: 137–8).^1^ In most models we employ county-level fixed effects to control for unobserved heterogeneity, such as county-level variations in the disease environment, which may have been significant (see, for example, county-level maps for 1910 in Dribe et al. 2020). We also cluster standard errors at the county level.

Independent variables, which we observe in Census A prior to the observation of children’s mortality, include dummy variables for residence type (rural, urban, population 2,500–9,999, urban 10,000–24,999, urban 25,000–99,999, urban 100,0000 plus); mother’s age group; age difference between spouses, couple’s nativity (native born or born in Germany, Ireland, Great Britain, Canada, or other foreign country), couple’s literacy, and wealth decile. We construct separate models using couples’ real property wealth decile in Census A (available in the 1850, 1860, and 1870 censuses), personal property wealth decile (available only in the 1860 and 1870 censuses), and total property wealth decile (also available only in the 1860 and 1870 censuses).

For each dataset, we construct a model for the entire nation and a model for each of the three major census regions, Northeast, Midwest, and South (the West census region included too few observations for consistent analysis). We also construct national models for the rural, agricultural population (children of fathers whose occupation was “farmer” or “farm laborer”) and the urban, nonagricultural population. In these two models, we shift the area fixed effects from the county level to the state economic area (SEA) level to ensure enough within-unit variation. SEAs, which were defined by the 1950 Census Bureau, are aggregations of two or more contiguous counties with similar economic orientations and are a level of geographic aggregation between counties and states. In the 1870–80 MLP dataset, for example, couples resided in 2,239 counties, 439 SEAs, and 48 states or territories.

In all models, couples with no reported wealth are the reference group.^2^ Because we are modeling the proportion of each couple’s children dying using OLS and weighting the results by the number of children at risk in Census A, positive coefficients represent the increase in the proportion of children dying relative to the reference group, while negative coefficients represent the decrease in the proportion dying, all else being equal.

## Results

Full model results are shown in the Online Appendix and confirm most expectations, such as higher mortality in urban areas. Here, we limit our discussion to the relationship between parents’ wealth decile and child mortality.

**Figure 1.**
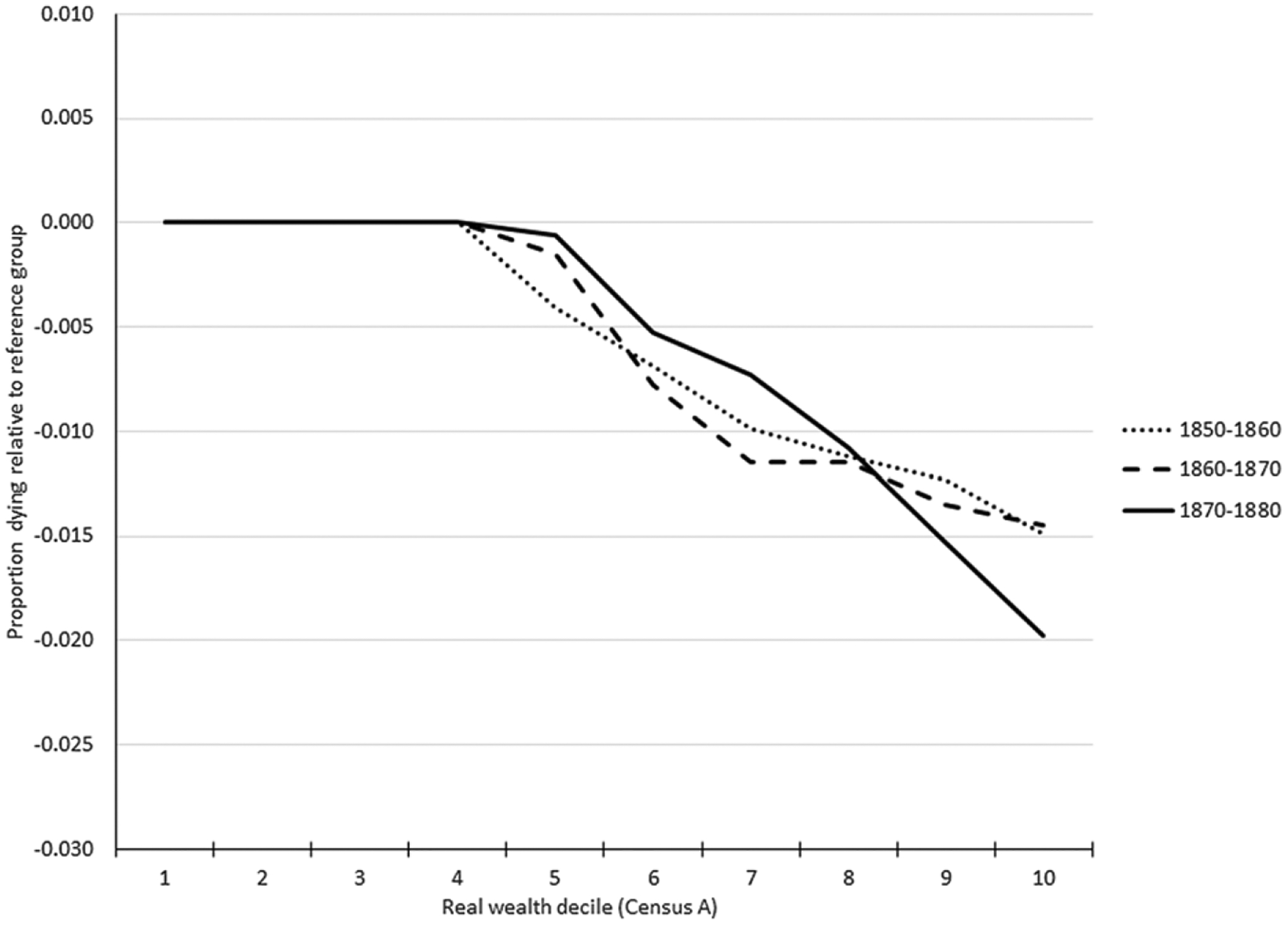
Child mortality by parents’ real wealth decile. Source: See Table 2.

**Figure 2.**
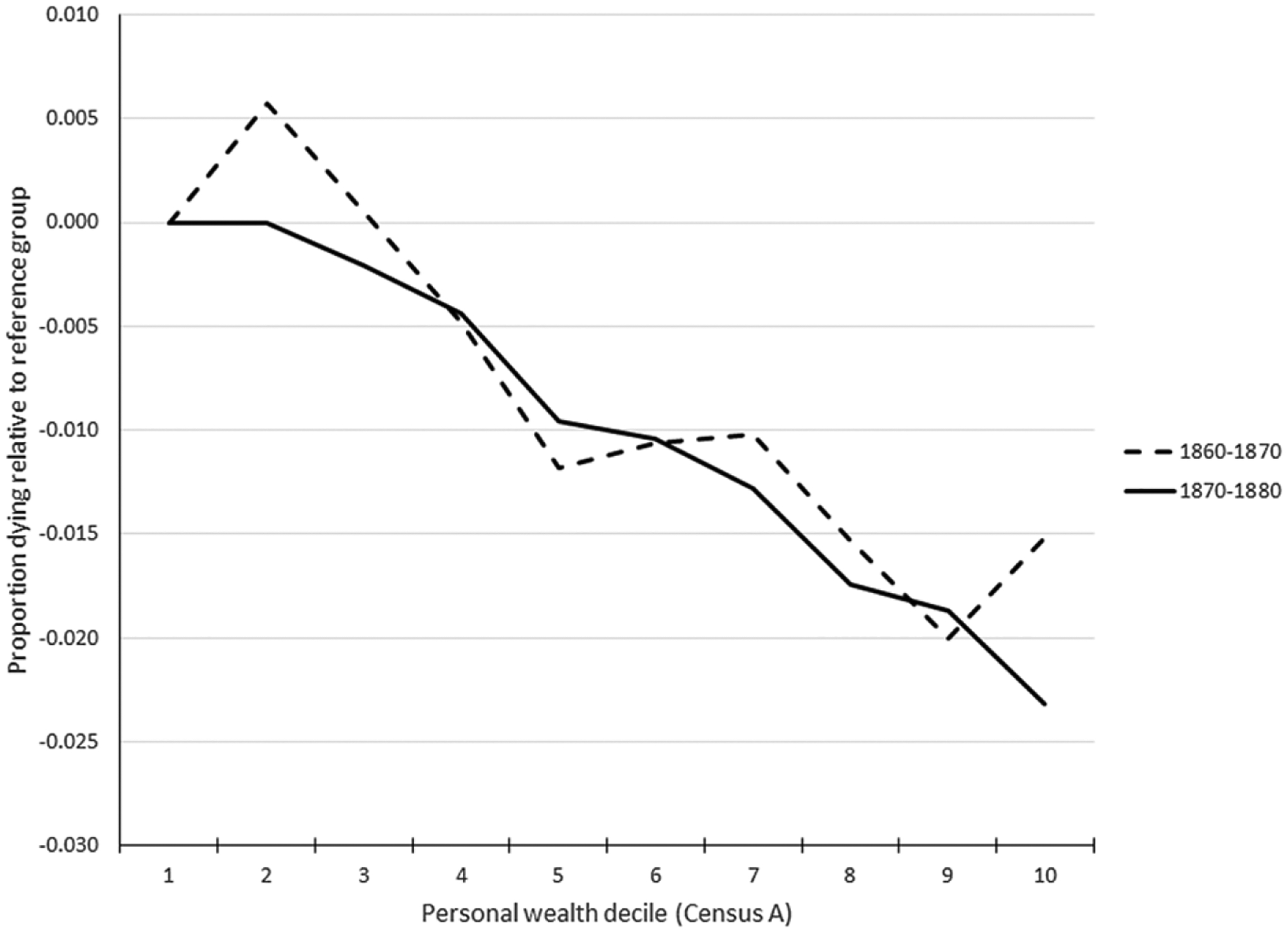
Child mortality by parents’ personal wealth decile. Source: See Table 2.

**Figure 3.**
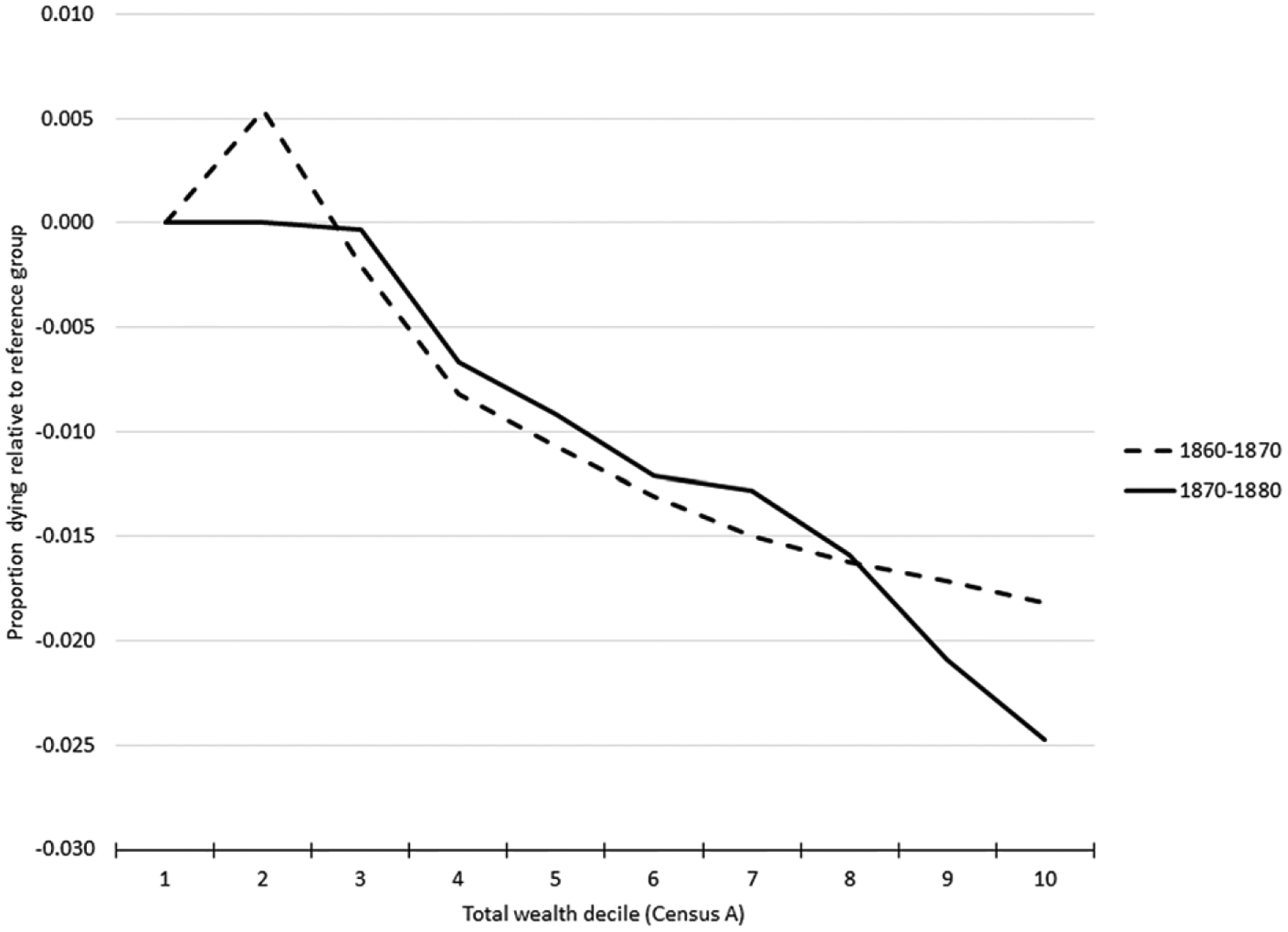
Child mortality by parents’ total wealth decile. Source: See Table 2.

Figures 1, 2 and 3 display the overall national results for the three wealth variables (real, personal, and total property) for couples in each period. Figure 1 plots the relationship between couples’ real-estate wealth and child mortality. The patterns are similar across the three periods. Approximately 40–50% of couples had no real estate wealth. In the upper half of the wealth distribution, we see a remarkably linear negative relationship between wealth and child mortality, with children of parents in each higher decile experiencing progressively lower mortality relative to the reference group of children born to parents with no real estate wealth, and lower mortality than the children born to parents in the next lower decile.^3^ Children of couples in the highest real estate wealth decile experienced about 1.5% fewer deaths in the 1850–60 and 1860–70 panel datasets relative to children of couples with no real wealth. In the 1870–80 panel dataset, the difference was 2% fewer. Since ten-year mortality for children in the 1–3-year age group averaged about 10% in the three panel studies, this was approximately equivalent to a 15–20% lower rate of mortality.

Figures 2 and 3 indicate very similar patterns between personal estate wealth and child mortality and total estate wealth and child mortality. The plots for personal and total wealth (couples combined real and personal wealth) for couples in the 1860–70 panel dataset suggest that mortality was modestly higher for the children with the lowest non-zero decile of wealth relative to children of no wealth couples.^4^ The top 20% wealthiest families experienced about 2 percentage points lower child mortality than the bottom 20%, which is a non-trivial magnitude given the mean percentage of children aged 1–3 dying within 10 years, which was 11% in the 1860–70 dataset and 9% in 1870–80 dataset (see Table 2).

**Figure 4.**
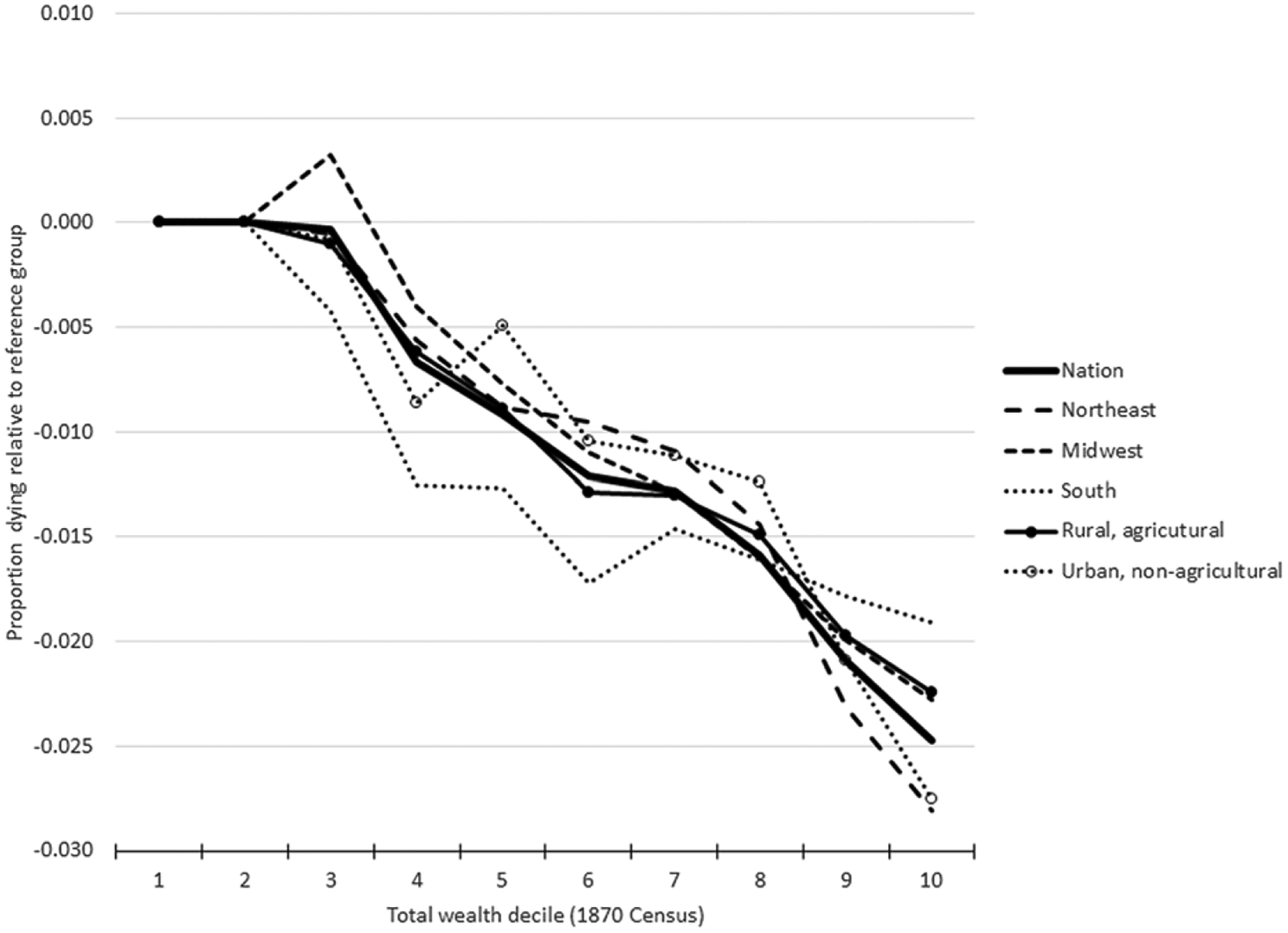
Child mortality, 1870–80, by parents’ total wealth decile. Source: See Table 2.

Turning to the regional models, we see similar results. Tables 3, 4 and 5 show the results for couples’ three measures of wealth in the three datasets (full model results can be found in the Online Appendix). In all contexts – whether national or regional, in models limited to the urban nonfarm population or to the rural farm population, or in models for the three periods studied – we find a negative gradient between wealth and child mortality. Although children born to parents in a higher wealth decile will occasionally have modestly higher mortality than children born to parents in the immediately adjacent lower decile, the gradients we find are remarkably smooth. Interestingly, despite the different contexts, the patterns are quite similar across regions. The gradient was somewhat steeper in the Northeast census region than in the South census region and in the urban non-agricultural population than in the rural agricultural population (see Figure 4, which plots the relationship between total wealth and child mortality by region in the 1870–80 interval, which was less likely to have been influenced by the American Civil War between 1861 and 1865). In terms of magnitudes, the difference between the top-10% and bottom-10% of the total wealth distribution range in 1870–80 was 1.9 percentage points in mortality in the South and 2.8 percentage points in the Northeast. The corresponding differences were about 2.2 percentage points in rural agricultural occupations and 2.8 percentage points in urban non-agricultural occupations. These are minor differences given the dramatically different contexts. It clearly demonstrates the universal negative association between wealth and child mortality in the United States in this period.

## Conclusion

Although a socioeconomic gradient in health and mortality appears universal in modern societies, its historical origins remain unclear. In the United States, analysis of the historical relationship between socioeconomic status and mortality has been limited by poor data. The nation’s death registration was first established in 1900 and not completed until 1933. As a result, most historical studies of the mortality gradient have focused on the turn of the twentieth century, when children ever born and children surviving data collected by the 1900 and 1910 censuses allow the measurement of child mortality and the testing of hypotheses. Even in that context, the lack of income and wealth data limits the investigation to occupation and homeownership. Although modest gradients have been found among children born to parents in different occupational groups and between children born to parents who did and did not own their own homes, heterogeneity of income and wealth within occupation groups may obscure the existence of a larger mortality gradient.

The main contribution of this study is to leverage the recently created IPUMS MLP links for the censuses 1850–60, 1860–70, and 1870–80 to examine the relationship between parental wealth and child mortality. Our findings indicate the presence of a negative relationship between the wealth of parents and the mortality of children already in the first period 1850–60. We find a remarkably smooth negative gradient, with children born to parents at each higher wealth decile experiencing lower mortality than the children born to parents at lower wealth deciles. The relationship was apparent both when using real estate wealth and personal wealth.

The wealth gradient in child mortality was also clearly evident within each of the three major census regions, among both the urban non-agricultural and rural agricultural populations, and was quite consistent across the three decades examined (1850–60, 1860–70, and 1870–80). The estimated magnitudes were significant. The difference in child mortality between the top-10% and bottom-10% of total wealth was 1–3 percentage points. Overall, however, only about 10% of children aged 1–3 died in the following ten-year period, so these differences represented about a 10–30% higher risk of death among children whose parents were in the lowest decile of wealth relative to children whose parents were in the highest decile of wealth.

These results suggest that the mortality gradient in the United States was long standing, at least for infants and children. Although this conclusion is supportive of fundamental cause theory (Link and Phelan 1995, 1996), historical demographers might find it surprising. The period between 1850 and 1880 was prior to the mortality transition, which commenced in the United States in the 1870s, and the wide-spread acceptance of the germ theory of disease. Access to medical care was in most cases of dubious value and public health efforts focused only on the elimination of noxious odors and visually unclean water (Duffy 1992). Eighteenth and nineteenth-century Americans, judging by their heights, appear to have been well fed, although a decline in heights of about one inch on average during the antebellum era may have been associated with growing inequality (Steckel 2009; Carson 2013). Evidence for U.S. passport applicants, for example, suggests that a gap between elite and non-elite heights were growing between 1800 and 1860 (Sunder 2013). But higher net nutritional status provided little protection from acute infectious diseases, which were the primary killers of adults and children. Moreover, residential segregation by class and race was low. According to Daniel Scott Smith, it was an era when “one was killed, so to speak, by his or her neighbors” (1983).

Nonetheless, parental wealth can affect the mortality of children through several potential pathways that we are unable to measure directly, and those pathways may have included some role for differentials in nutrition, as well as roles for differences in health care, environment, and parental behaviors, such as breastfeeding and child spacing. Although knowledge about disease was rudimentary in the mid-nineteenth century, the early sanitation movement may have led to new forms of cleanliness and domestic hygiene among wealthier groups that were advantageous to child survival (Bushman and Bushman 1988; Tomes 1990; Duffy 1992). Our results are consistent with the hypothesis that wealth allowed parents to buy modest survival advantages for their children, but not to distinguish whether those advantages were related to better living conditions, housing, environment, health care and nutrition, or possibly to different parental behavior and lifestyle not associated with wealth or income as such but with higher social status.

While census data are invaluable to estimating the relationship between wealth and child mortality across the entire United States during a period for which we lack civil registration data, they are not as helpful when trying to assess the mechanisms. In future research, other sources could be explored to get more in-depth knowledge about the likely pathways between parental wealth and child mortality.

## Supplementary Material

appendix

## Figures and Tables

**Table 1.** Comparison of the cross-sectional and linked datasets

Dataset	Cross-sectional complete-count			Linked MLP		
Census year(s)	1850	1860	1870	1850–1860	1860–1870	1870–1880
Fertility (number)						
Own children	3.85	3.62	3.59	4.19	3.86	3.77
Own children 0–4 years	1.58	1.59	1.56	1.64	1.62	1.59
Mother’s age group (%)						
20–24	16.9	17.1	16.2	14.0	15.1	14.6
25–29	27.1	27.6	26.5	25.8	26.5	26.1
30–34	23.5	24.3	23.6	24.5	25.0	24.2
35–39	18.0	17.6	19.0	19.7	18.9	19.8
40–44	10.9	10.2	11.1	12.2	11.3	11.8
45–49	3.7	3.2	3.6	3.9	3.4	3.6
Age difference from spouse (years)	−4.7	−4.9	−5.3	−4.5	−4.8	−5.1
Father’s wealth (average dollars)						
Real estate wealth	$ 1 051	$ 1 954	$ 1 950	$ 1 274	$ 2 257	$ 2 258
Personal estate wealth		$ 1 414	$ 921		$ 1 462	$ 1 030
Combined estate wealth		$ 3 368	$ 2 871		$ 3 719	$ 3 288
Father’s occupation group (%)						
Professional, Technical	2.9	2.8	2.6	2.7	2.8	2.6
Farmers (owners and tenants)	51.8	40.4	42.1	58.3	45.2	45.9
Managers, officials, and proprietors	5.4	5.9	6.5	5.2	6.0	6.5
Clerical and Kindred	0.2	0.5	1.0	0.2	0.5	0.9
Sales workers	0.8	1.2	1.6	0.7	1.0	1.5
Craftsmen	17.1	17.4	15.2	16.0	16.9	14.8
Operatives and apprentices	8.7	7.5	10.6	7.9	7.0	10.1
Service workers	0.5	1.0	1.1	0.4	0.8	0.9
Farm laborers	0.2	3.2	6.3	0.1	2.9	5.7
Laborers	11.6	11.1	10.0	8.0	8.6	8.0
Nonoccupational response	0.7	9.0	3.0	0.5	8.4	3.0
Literacy of couple (%)						
Illiterate	17.2	13.9	19.2	15.2	11.9	17.4
Literate (both partners can read and write)	82.8	86.1	80.8	84.9	88.1	82.6
Nativity of couple (%)						
United States	82.4	71.1	67.7	89.5	76.8	71.6
German	5.8	10.5	12.7	2.8	7.7	11.0
Ireland	7.6	12.7	12.3	4.5	10.1	10.9
Great Britain	4.0	5.1	5.3	3.5	5.4	5.6
Canada	1.4	2.0	3.0	0.9	1.8	2.7
Other foreign born	1.1	3.4	4.6	0.6	2.1	3.4
Region of residence (%)						
Northeast	41.3	36.5	33.1	43.4	39.4	34.9
Midwest	29.7	36.7	41.8	29.7	38.3	42.7
South	28.6	25.3	22.6	26.7	21.1	20.3
West	0.4	1.5	2.6	0.2	1.2	2.2
Urban/rural residence type (%)						
Rural	85.5	79.4	75.5	90.2	83.6	79.2
Urban, less than 10,000 pop.	2.8	4.4	4.8	2.4	4.1	4.8
Urban, 10,000–24,999 pop.	2.5	2.8	3.8	2.0	2.7	3.5
Urban, 25,000–99,999 pop.	4.2	3.9	4.5	2.7	3.2	3.9
Urban, 100,000+ pop.	5.1	9.6	11.4	2.6	6.4	8.6
Children age 10–19 in second census linked to a child age 0–9 in first census (%)				86.4	83.2	84.1
Number of couples	1,507,251	2,121,540	2,476,520	627,883	887,988	1,226,756
Estimated number of couples surviving to the next census	1,141,738	1,524,802	1,971,823			
Estimated number of couples surviving and enumerated in both censuses	1,018,990	1,352,728	1,781,897			
Percentage of “linkable” couples linked				61.6	65.6	68.8

Sources: IPUMS 1850, 1860, and 1870 complete-count datasets (Ruggles et al. 2021) and IPUMS MLP datasets (Helgertz et al. 2020).

Note: Universe: The cross-sectional dataset includes all currently married white couples with women age 20–49 and with one or more children age 0–4 in the household in the IPUMS complete-count datasets with spouses aged 25–59. Couples in the linked datasets include currently married white couples with women age 20–49 in the first of the two censuses with one or more children age 0–4in the first census.

Displayed values are the percentages of cases in each group unless labeled otherwise. Values shown for the linked datasets are from the first of two linked censuses (Census A). The one exception is the proportion of children aged 10–19 in the second of the two linked censuses (Census B) who were linked back to a child age 0–9 in Census A. The number of surviving couples were estimated using the 10-year survival probabilities in Hacker (2010) for the native-white population. Because the survival probability of the foreign-born population was likely lower, the true number of estimated couples surviving to the next census was likely somewhat lower. The estimated number of surviving and enumerated couples relied on the overall net census undercount estimates for both sexes combined reported in Hacker (2013). Because the net undercount included a small but unknown number of double-counted couples, the true number of couples who were potentially linkable in both censuses was likely somewhat lower.

**Table 2.** Descriptive statistics, proportion of children aged 1–3 dying in intercensal interval

	1860–1870		1860–1870		1870–1880	
	Proportion dying	N	Proportion dying	N	Proportion dying	N
Total wealth tercile						
First			0.124	239,655	0.099	314,996
Second			0.108	268,644	0.086	367,219
Third			0.107	257,840	0.078	343,706
Father’s occupation						
Professional, technical	0.098	14,454	0.115	20,135	0.084	24,914
Farmers (owners and tenants)	0.086	323,285	0.103	341,128	0.077	469,059
Managers, officials, and proprietors	0.105	27,819	0.118	43,670	0.090	64,050
Clerical and kindred	0.110	1,023	0.108	3,406	0.087	9,158
Sales workers	0.107	3,553	0.120	7,748	0.093	14,727
Craftsmen	0.105	88,508	0.121	129,973	0.098	149,237
Operatives and apprentices	0.107	42,970	0.121	54,458	0.099	105,900
Service workers	0.125	1,902	0.125	6,027	0.107	9,555
Farm laborers	0.106	752	0.119	23,585	0.088	64,555
Laborers	0.112	45,887	0.131	70,077	0.108	84,286
Nonoccupational response	0.097	2,795	0.111	65,932	0.085	30,480
Literacy						
Illiterate	0.091	89,397	0.111	94,136	0.092	178,875
Literate	0.095	463,551	0.113	672,003	0.086	847,046
Nativity						
Born in the United States	0.094	487,304	0.113	556,315	0.085	702,528
Born in Canada	0.088	5,242	0.102	14,258	0.087	28,450
Born in Great Britain	0.094	18,483	0.106	40,075	0.087	52,280
Born in Ireland	0.096	23,728	0.119	80,112	0.097	106,062
Born in Germany	0.102	14,979	0.117	57,465	0.099	101,711
Other Foreign Born	0.101	3,212	0.097	17,914	0.083	34,890
Region						
Northeast	0.100	223,545	0.125	285,736	0.108	853,592
Midwest	0.091	168,075	0.102	301,696	0.090	909,677
South	0.090	160,280	0.112	168,002	0.094	546,404
West	0.107	1,048	0.107	10,705	0.092	35,335
Urban/rural residence type						
Rural	0.092	501,365	0.110	640,822	0.083	816,024
Urban, less than 10,000 pop.	0.114	13,064	0.117	31,641	0.091	48,082
Urban, 10,000–24,999 pop.	0.116	10,204	0.125	19,663	0.099	35,527
Urban, 25,000–99,999 pop.	0.125	14,376	0.119	24,714	0.106	39,042
Urban, 100,000+ pop.	0.124	13,939	0.143	49,299	0.117	87,246
Total	0.094	552,948	0.113	766,139	0.087	1,025,921

Source: See Table 1.

Notes: Universe: Currently married white couples with women aged 20–49 with spouses aged 25–59 and with one or more children aged 1–3 in the household after adjustments for undercounting as described in text.

Results were weighted by the number of couples’ children aged 1–3. In 1860, the total wealth terciles for couples in the model were $0–$299, $300–$1699, and $1700 and above. In 1870 they were $0–$299, $300–$1899, and $1900 and above.

**Table 3.** OLS regression, child mortality in intercensal interval. Models with parents’ real estate wealth

	Linked Couples in the 1850–1860 IPUMS MLP Dataset					
	Nation	Northeast	Midwest	South	Non-Agricultural, urban	Agricultural occupations, rural
Parents’ real estate wealth						
Decile 1 – no wealth (ref.)	0.000	0.000	0.000	0.000	0.000	0.000
Decile 2 – no wealth (ref.)	0.000	0.000	0.000	0.000	0.000	0.000
Decile 3 – no wealth (ref.)	0.000	0.000	0.000	0.000	0.000	0.000
Decile 4 – no wealth (ref.)	0.000	0.000	0.000	0.000	0.000	0.000
Decile 5	−0.004 **	−0.004	0.000	−0.006 **	0.001	−0.001
Decile 6	−0.007 ***	−0.007 **	−0.004	−0.008 **	−0.005	−0.005 **
Decile 7	−0.010 ***	−0.012 ***	−0.006 *	−0.009 ***	−0.016 *	−0.006 ***
Decile 8	−0.011 ***	−0.017 ***	−0.003	−0.010 **	0.003	−0.008 ***
Decile 9	−0.012 ***	−0.017 ***	−0.011 ***	−0.004	0.006	−0.007 **
Decile 10	−0.015 ***	−0.025 ***	−0.007 *	−0.004	−0.014 *	−0.005 **
Number of couples	539,235	215,586	164,864	157,795	46,066	311,450
*R* ^2^	0.011	0.009	0.010	0.014	0.007	0.005
	Linked Couples in the 1860–1870 IPUMS MLP Dataset					
	Nation	Northeast	Midwest	South	Non-Agricultural, urban	Agricultural occupations, rural
Parents’ real estate wealth						
Decile 1 – no wealth (ref.)	0.000	0.000	0.000	0.000	0.000	0.000
Decile 2 – no wealth (ref.)	0.000	0.000	0.000	0.000	0.000	0.000
Decile 3 – no wealth (ref.)	0.000	0.000	0.000	0.000	0.000	0.000
Decile 4 – no wealth (ref.)	0.000	0.000	0.000	0.000	0.000	0.000
Decile 5	−0.002	0.000	0.001	−0.005 *	0.000	−0.001
Decile 6	−0.008 ***	−0.008 **	−0.006 **	−0.009 ***	−0.007 *	−0.006 ***
Decile 7	−0.012 ***	−0.012 ***	−0.011 ***	−0.012 ***	−0.009 *	−0.010 ***
Decile 8	−0.012 ***	−0.012 ***	−0.011 ***	−0.013 ***	−0.007	−0.009 ***
Decile 9	−0.014 ***	−0.016 ***	−0.012 ***	−0.013 ***	−0.009	−0.011 ***
Decile 10	−0.015 ***	−0.025 ***	−0.009 ***	−0.008 *	−0.014 ***	−0.011 ***
Number of couples	750,469	277,673	297,968	164,668	115,648	358,896
*R* ^2^	0.009	0.006	0.006	0.016	0.007	0.005
	Linked Couples in the 1860–1870 IPUMS MPL Dataset					
	Nation	Northeast	Midwest	South	Non-Agricultural, urban	Agricultural occupations, rural
Parents’ real estate wealth						
Decile 1 – no wealth (ref.)	0.000	0.000	0.000	0.000	0.000	0.000
Decile 2 – no wealth (ref.)	0.000	0.000	0.000	0.000	0.000	0.000
Decile 3 – no wealth (ref.)	0.000	0.000	0.000	0.000	0.000	0.000
Decile 4 – no wealth (ref.)	0.000	0.000	0.000	0.000	0.000	0.000
Decile 5	−0.001	0.002	0.001	−0.005 *	−0.010	−0.002
Decile 6	−0.005 ***	−0.002	−0.004 *	−0.011 ***	−0.006 *	−0.007 ***
Decile 7	−0.007 ***	−0.004 *	−0.009 ***	−0.007 **	−0.003	−0.007 ***
Decile 8	−0.011 ***	−0.010 ***	−0.012 ***	−0.010 ***	−0.007 *	−0.010 ***
Decile 9	−0.015 ***	−0.014 ***	−0.017 ***	−0.010 ***	−0.017 ***	−0.014 ***
Decile 10	−0.020 ***	−0.024 ***	−0.019 ***	−0.013 ***	−0.020 ***	−0.017 ***
Number of couples	1,027,252	341,499	443,895	218,777	201,161	538,600
*R* ^2^	0.011	0.008	0.008	0.017	0.008	0.007

Source: See Table 1.

Notes: The dependent variable is the proportion of children age 1–3 in Census A dying prior to Census B. Results are weighted by the number of children at risk of death. The models for the nation and each of the three major regions employ county-level fixed effects. The models for the nonagricultural, urban, and the agricultural, rural populations employ SEA-level fixed effects.

* *p* < 0.05.

** *p* < 0.01.

*** *p* < 0.005.

**Table 4.** OLS regression, child mortality in intercensal interval. Models with parents’ personal estate wealth

	Linked Couples in the 1860–1870 IPUMS MLP Dataset					
	Nation	Northeast	Midwest	South	Non-Agricultural, urban	Agricultural occupations, rural
Parents’ personal estate wealth						
Decile 1 – no wealth (ref.)	0.000	0.000	0.000	0.000	0.000	0.000
Decile 2	0.006 **	0.004	0.007 *	0.008	0.007	0.002
Decile 3	0.001	0.002	0.001	−0.002	−0.004	0.000
Decile 4	−0.005 **	−0.004	−0.004	−0.008 *	−0.005	−0.004
Decile 5	−0.012 ***	−0.013 ***	−0.008 **	−0.016 ***	−0.015 ***	−0.011 ***
Decile 6	−0.011 ***	−0.009 **	−0.009 **	−0.013 **	0.000	−0.009 ***
Decile 7	−0.010 ***	−0.013 ***	−0.007 *	−0.010 *	−0.007	−0.010 ***
Decile 8	−0.015 ***	−0.017 ***	−0.013 ***	−0.015 ***	−0.016 *	−0.013 ***
Decile 9	−0.020 ***	−0.024 ***	−0.015 ***	−0.020 ***	−0.020 ***	−0.020 ***
Decile 10	−0.015 ***	−0.026 ***	−0.010 **	−0.012 **	−0.021 ***	−0.015 ***
Number of couples	750,469	277,673	297,968	164,668	115,648	358,896
*R* ^2^	0.009	0.006	0.006	0.016	0.008	0.006
	Linked Couples in the 1870–1880 IPUMS MLP Dataset					
	Nation	Northeast	Midwest	South	Non-Agricultural, urban	Agricultural occupations, rural
Parents’ personal estate wealth						
Decile 1 – no wealth (ref.)	0.000	0.000	0.000	0.000	0.000	0.000
Decile 2 – no wealth (ref.)	0.000	0.000	0.000	0.000	0.000	0.000
Decile 3	−0.002	−0.003	−0.002	−0.002	−0.004	−0.002
Decile 4	−0.004 ***	−0.004	−0.003	−0.008 **	−0.003	−0.003
Decile 5	−0.010 ***	−0.004 *	−0.010 ***	−0.016 ***	−0.008 **	−0.010 ***
Decile 6	−0.010 ***	−0.009 **	−0.010 ***	−0.013 ***	−0.011 **	−0.009 ***
Decile 7	−0.013 ***	−0.013 ***	−0.012 ***	−0.014 ***	−0.011 ***	−0.011 ***
Decile 8	−0.017 ***	−0.017 ***	−0.018 ***	−0.018 ***	−0.013 ***	−0.016 ***
Decile 9	−0.019 ***	−0.022 ***	−0.018 ***	−0.013 ***	−0.002	−0.018 ***
Decile 10	−0.023 ***	−0.029 ***	−0.021 ***	−0.017 ***	−0.026 ***	−0.021 ***
Number of couples	1,027,252	341,499	443,895	218,777	201,161	538,600
*R* ^2^	0.011	0.009	0.008	0.017	0.008	0.007

Source: See Table 1.

Notes: See Table 3.

**Table 5.** OLS regression, child mortality in intercensal interval. Models with parents’ total estate wealth

	Linked Couples in the 1860–1870 IPUMS MLP Dataset					
	Nation	Northeast	Midwest	South	Non-Agricultural, urban	Agricultural occupations, rural
Parents’ total estate wealth						
Decile 1 – no wealth (ref.)	0.000	0.000	0.000	0.000	0.000	0.000
Decile 2	0.005 **	0.005	0.008 *	0.002	0.002	0.005
Decile 3	−0.002	−0.002	0.003	−0.011 *	−0.008	0.000
Decile 4	−0.008 ***	−0.010 ***	−0.003	−0.013 ***	−0.010 **	−0.007 **
Decile 5	−0.011 ***	−0.009 **	−0.008 *	−0.014 ***	−0.013 **	−0.011 ***
Decile 6	−0.013 ***	−0.016 ***	−0.009 ***	−0.012 **	−0.014 ***	−0.010 ***
Decile 7	−0.015 ***	−0.017 ***	−0.010 ***	−0.020 ***	−0.014 *	−0.013 ***
Decile 8	−0.016 ***	−0.017 ***	−0.012 ***	−0.020 ***	−0.014 *	−0.015 ***
Decile 9	−0.017 ***	−0.021 ***	−0.011 ***	−0.021 ***	−0.019 ***	−0.015 ***
Decile 10	−0.018 ***	−0.032 ***	−0.011 ***	−0.013 **	−0.018 ***	−0.017 ***
Number of couples	750,469	277,673	297,968	164,668	115,648	358,896
*R* ^2^	0.010	0.006	0.006	0.016	0.007	0.006
	Linked Couples in the 1870–1880 IPUMS MLP Dataset					
	Nation	Northeast	Midwest	South	Non-Agricultural, urban	Agricultural occupations, rural
Parents’ total estate wealth						
Decile 1 – no wealth (ref.)	0.000	0.000	0.000	0.000	0.000	0.000
Decile 2 – no wealth (ref.)	0.000	0.000	0.000	0.000	0.000	0.000
Decile 3	0.000	−0.001	0.003	−0.004	−0.001	−0.001
Decile 4	−0.007 ***	−0.006 **	−0.004 *	−0.013 ***	−0.009 ***	−0.006 ***
Decile 5	−0.009 ***	−0.009 ***	−0.008 ***	−0.013 ***	−0.005	−0.009 ***
Decile 6	−0.012 ***	−0.010 ***	−0.011 ***	−0.017 ***	−0.010 ***	−0.013 ***
Decile 7	−0.013 ***	−0.011 ***	−0.013 ***	−0.015 ***	−0.011 ***	−0.013 ***
Decile 8	−0.016 ***	−0.015 ***	−0.016 ***	−0.016 ***	−0.012 ***	−0.015 ***
Decile 9	−0.021 ***	−0.023 ***	−0.020 ***	−0.018 ***	−0.021 ***	−0.020 ***
Decile 10	−0.025 ***	−0.028 ***	−0.023 ***	−0.019 ***	−0.028 ***	−0.022 ***
Number of couples	1,027,252	341,499	443,895	218,777	201,161	538,600
*R* ^2^	0.011	0.009	0.008	0.017	0.008	0.007

Source: See Table 1.

Notes: See Table 3.

## Footnotes

1 Because our dependent variable was limited in most cases to just a few values (0.0 and 1.0 for couples with one child at risk, 0.0, 0.5, and 1.0 for couples with two children at risk, and 0.0, 0.33, 0.66, and 1.0 for three children), heteroskedasticity may bias estimates of standard errors. Fortunately, coefficients are unbiased and the large number of cases in the IPUMS MLP datasets ensures that standard errors were very low. Logistic regression models with the mortality or survival of each child as the dependent variable represent a possible alternative to OLS without the risk of potential bias from heteroskedasticity. We decided to rely on weighted OLS regression for several reasons. First, beginning with Preston and Haines (1991), a large majority of the published research on child mortality in the historical United States (e.g., Preston et al. 1994; Hacker and Haines 2005; Dribe et al. 2020, and several of the articles published in this special issue of *Social Science History*) have relied on weighted OLS at the couple level. Our OLS results, therefore, will be directly comparable with existing scholarship. Second, our method of forcing links between unmatched children, estimating the number of children missed by the first census, and estimating the number of children dying, is currently conducted at the couple level, using only the number and ages of each couple’s children in each census. We are currently working to shift our estimation methods to the individual child level while incorporating additional variables (names, birthplaces, and ages of siblings) to obtain data to support child-level analyses.

2 In some models, no-wealth couples composed about 10% of the couples in the model, while in other models, such as the model of the relationship between couples’ real estate wealth and fertility in the 1850–60 panel dataset, they represented more than 50% of couples in the model. The reference group of no-wealth couples could therefore represent more than one wealth decile. In these cases, we show the reference group coefficient value (0.0) for each applicable decile. Coefficients are shown for all other deciles with couples reporting nonzero wealth, even if rounding and truncation of wealth information – typically at values that were multiples of $500 and $1,000 – meant that the percentage of couples in different deciles was over or under 10% of couples in the model. Occasionally truncation and rounding produced erratic estimates, especially in the first decile with reported wealth information, which typically contained well under 10% of the couples in the model.

3 We did not test each coefficient for differences with other coefficients.

4 We also find a similar result – the coefficient was positive for the mortality of children born to couples in the first wealth decile with reported wealth above zero – in a few of the region-specific models. In most of these models the coefficient was small and not statistically significant at the 0.05 level. We believe this result is largely spurious, a result of small numbers of cases in the first decile with reported wealth and small selection biases in the quality of the enumeration between couples who reported no wealth and couples who reported very low levels of wealth (e.g., $100 of personal wealth)
