# Supplementary material for: Wealth and Child Mortality in the Nineteenth-Century United States: Evidence from Three Panels of American Couples, 1850–1880
Source: Soc Sci Hist. Author manuscript; Available in PMC 2024 Oct 1. (PMC11415258; doi:10.1017/ssh.2023.12)
Supplement: appendix [file NIHMS1971707-supplement-appendix.pdf]

Wealth and Child Mortality in the Nineteenth-Century United States
Online Appendix

J. David Hacker, Martin Dribe, and Jonas Helgertz[1]
May 9, 2023

In this appendix we provide more detail on how we measure child mortality and discuss the potential impact of our assumptions on the results. We also provide complete regression results for models discussed in the main text and regression results when using inverse propensity weights that attempt to compensate for the modest selection biases in the linked datasets documented in Table 1 of the main text.

Measuring Child Mortality

To estimate child mortality, we begin by restricting the analytical dataset to married couples linked between the 1850-1860, 1860-1870, and 1870-1880 censuses who had one or more coresident own children in the first of the two linked censuses (hereafter, Census "A"). We then determined the number of those children who were enumerated in the second of the two censuses (hereafter, Census "B") using the links provided by the IPUMS MLP project. Finally, we assumed that children in Census A who were not linked to a child in Census B died in the ten-year interval between the two censuses. Dividing the number of children dying by the number of children at risk in Census A resulted in the proportion of each couple's children at risk children dying in the ten-year interval.

[1] J. David Hacker, University of Minnesota, Department of History and Minnesota Population Center, hacker@umn.edu; Martin Dribe, Lund University, Department of Economic History and Centre for Economic Demography, Martin.Dribe@ekh.lu.se; Jonas Helgertz, University of Minnesota, Minnesota Population Center and Lund University, Department of Economic History and Centre for Economic Demography, helgertz@umn.edu.

This basic approach is subject to several sources of error, which we attempted to minimize by restricting our analysis to children in selected age groups and by making a few corrective adjustments to the data. Children who were still living in Census B but who had left their parents' homes were less likely to be linked between the two censuses. If not linked, these children will appear in our analysis to be deceased. Because the propensity for children to leave their parents' households likely varied by the wealth and other characteristics of their parents, the unobserved departure of children from their parents' homes could bias our results. Fortunately, the median age at leaving home in the mid nineteenth century United States was over 25 years for both men and women (Steckel 1996) and very few children with two surviving parents appeared to have left their parents' households before aged 14 relative to the number of those children who died. By limiting our analysis to children aged 0-3 in Census A (corresponding to children aged 10-13 in Census B), we minimized this source of potential bias.

As shown in Table 1 of the main text, 16 percent of children aged 10-19 in the 1880 were not linked back to a child in aged 0-9 in the 1870 census. Similar percentages characterized the 1850-1860 and 1860-1870 panel datasets. These children reflected two other sources of potential bias: the under-enumeration of children in Census A (when these children were aged 0-9) or children who were present in both censuses but, for whatever reasons, were not linked in the IPUMS MLP data. Younger children appeared to have been at a much higher risk of being undercounted by nineteenth-century censuses than older children (Hacker 2013). Children present in Census A who were not enumerated by the census will bias the number of children at risk of death downwards and the estimated proportion dying in the intercensal interval upwards. We took two steps to minimize this bias. First, because our analysis indicated that children under the age of 1 were

approximately 4 times more likely to be missed by the census than other children, we decided to further restrict our mortality analysis to children aged 1-3 in Census A (corresponding to children aged 11-13 in Census B). Second, in cases where all children present in Census A were linked forward to a child in Census B, we considered the remaining children in Census B (who were not linked back to a child in Census A) to be evidence of an unenumerated child ten years before. In these cases (which represented about 1-in-3 of the 16% of couples with a child aged 10-19 in Census B who were not linked back to Census A), we inflated the number of at-risk children in Census A at the year of birth (census year minus age) associated with the unlinked child in Census B.

About 2-in-3 of couples with children aged 10-19 in Census B who were not linked back to a child in Census A also had children in Census A who were not linked forward to a child in Census B. In the specific case of a couple with one child in Census A who was not linked forward and one child in Census B who was not linked backward, there were two likely possibilities: (1) the unlinked child in Census A died in the interval and the unlinked child in Census B was undercounted in Census A, or (2) these two children were the same child but who were not linked, for many possible reasons, by the IPUMS MLP project. Although the MLP linking algorithm linked a large majority of children present in Census B back to Census A, errors in the data (e.g., an incorrect age, sex, race, birthplace, or poorly spelled first name in either census or a combination of various errors) could result in a failure to identify all true links. This failure represented a third source of error in our mortality estimates. Examination of the original manuscript returns was often helpful in determining whether these children should have been linked. Unfortunately, the large number of cases in our dataset precluded a case-by-case assessment. We sampled several dozen linked couples with unlinked children in both censuses who shared the same

approximate year of birth. In almost all cases, the evidence strongly suggested that the unlinked child in Census B was the same child in Census A. Our 1870-1880 panel dataset, for example, included the 1870 Dent County, Missouri couple Joab and Elizabeth Hobson, aged 43 and 38 respectively, who were linked to the 1880 Benton County, Missouri couple Joab and Elisabeth Hobson, aged 53 and 48. Joab's state of birth was listed as North Carolina and Elizabeth's as Kentucky in both censuses. Although residing in different Missouri counties, the MLP linking procedures determined that this was the same couple. In 1870, Joab and Elizabeth had three male children, named "A L," "A J," and "F S," aged 4, 4, and 1, all born in the state of Missouri, who were not linked forward to a child in the 1880 census. In 1880, Joab and Elizabeth had three male children, named "Abraham Hobson," "Andrew Hobson," and "Phillip Hobson," aged 14, 14, and 11, all born in Missouri, who were not linked backward to a child in the 1870 census. It seemed highly likely that these children were the same children in both censuses.

To cover a range of possibilities and to assess the impact of our assumptions on the results, we constructed two versions of our child mortality estimate. In version 1, our "high" mortality assumption, we assumed that all children aged 1-3 in Census A who were not linked forward to a child in Census B died in the ten-year interval between the two censuses and that all children aged 11-13 in Census B who were not linked back to a child in Census A were undercounted by the Census A. In version 2, which we considered our "preferred" estimate, children in Census B who were not linked back to a child in Census A and children in Census A who were not linked forward in Census B were assumed to have been linked to one another if their estimated years of birth (census year – age) were within plus or minus one year of each other. All remaining unlinked children in Census B were assumed to be undercounted in Census A.

We used the preferred estimate—which resulted in a mortality estimate closely approximating life table estimates for the period—in the main text. Further below we show the robustness of our findings by comparing the results using the high mortality estimate, labeled as the "alternative estimate" in the figure. We also compared results using different age groups of children in Census A (e.g., children aged 0-3, 1-4, 2-6, etc.). Overall, apart from much older age groups of children, who appeared to have been more likely to leave home, the results were remarkably robust to different age groupings and choice of mortality estimates.

Potential Bias from Census Under-Enumeration Errors

Census coverage errors by age are readily apparent in nineteenth-century data and have the potential to bias mortality results derived from the numbers of children present in the census by age. A simple distribution of the population by single years of age provides indirect evidence of under-enumeration of infants and young children. Given robust natural population growth rates in the nineteenth century (equivalent to about 3% per year in the period of this study) and the cumulative increase in the risk of mortality with age, we would typically expect more surviving children aged 0 in the cross-sectional census returns than children aged 1, more surviving children aged 1 than aged 2, and so on. Given published estimates of age-specific mortality rates (Hacker 2010), there should be about 15-20% more children aged 0 than aged 2 in the census.[2] There are significantly *fewer* children aged 0 (and

[2] This estimate assumes 3% more births in each subsequent year and L_0 and L_2 estimates for the 1860s for both sexes combined in Hacker (2010).

age 1) in each census between 1850 and 1870 than children aged 2, however. To some extent, this result could reflect the misreporting of children's age (e.g., an 11-month-old child was reported as being aged 1 instead of aged 0). The result may have also reflected short-term fluctuations in the size of birth cohorts. The age patterns are relatively consistent from census to census, however, strongly suggesting that the observed age patterns reflected the under-enumeration of infants and young children. Using back projection methods, Hacker (2013) estimated net census undercounts in the native-born white population by age and sex. Although he presented only the average net undercount by 5-year age groups (Hacker 2013), unreported results by single years of age indicated a rapid decline in under-counting between infancy and age 5. In the 1870 census, for example, the overall net undercount of white children in the census aged 0-4 was 10.5%. At individual ages, however, the percentages were 23.5% for children aged 0, 15.7% for children aged 1, 5.3% at age 2, 2.6% at age 3, and 0.7% at age 4. Admittedly, these results are fallible—based as they were on model life tables of unknown accuracy and back projection estimates from multiple censuses—but the differences are too large to be explained by different mortality assumptions. Undoubtedly, many infants and a few young children were under-counted in nineteenth-century U. S. censuses.

The IPUMS MLP project allowed us to estimate census under-enumeration by matching children across censuses. For married couples linked between two censuses, children aged 10-19 in Census B should be present in Census A, with a few rare exceptions. In our linked datasets, however, only 84% of children were linked back to the first census, suggesting that under-enumeration may have been as high as 16 percent. As discussed above, however, many of these unlinked children were enumerated in Census A but—for whatever reasons—were not linked by the IPUMS MLP project, which only identified linked

individuals when there was a very low probability of a type 1 error. Poorly spelled names or errors in age, sex, race, and birthplace could result in a failed link. We examined a large number of cases in which a couple in the MLP dataset had: (1) an unlinked child age x in Census A; and (2) an unlinked child age $x + 9$, $x + 10$, or $x + 11$ in Census B. In nearly all cases, we determined that these children were in high probability the same child (census data, unfortunately, are rarely definitive). Accordingly, we matched all unlinked children in Census B who shared the same year of birth (census year – age) or were within plus or minus one year of the same year of birth of an unlinked child in Census A. After matching, all remaining children in Census B who were not linked back to Census A were assumed to be undercounted in Census A at age $x – 10$ years relative to the unlinked child in Census B.

Figure A1 shows the resulting undercount estimates by age of child through age 5 in the three panel datasets (Census year A). As expected, the results indicated much higher rates of under-enumeration of infants than other children. Compared to children of other ages, children aged 0 were approximately four times more likely to be missed by the census. The results suggested that the enumeration of infants born to linked couples was modestly better in 1870 than in 1850 or 1860. Children aged 1 were somewhat more likely to have been missed than children aged 2 or 3, but the differences were modest. Given the high rate of under-enumeration among children aged 0 and the greater potential for differential under-enumeration across different groups of parents, we decided to focus our analysis on the mortality of children aged 1-3.

Potential Bias from the Unobserved Departure of Children from their Parents' Homes

Our method assumed that children present in Census A who could not be linked to Census B died in the interval. Because the IPUMS MLP project used a two-stage linking process that relaxed linking thresholds for household member of males linked in the first round (Helgertz et al. 2020), it was much easier to link children who remained in their parents' households between Censuses A and B than children who were no longer present. Children who survived the intercensal period but were no longer living with their parents were less likely to be linked, and if unlinked, would be interpreted as being deceased using our estimation method.

The potential for bias is greater for older children, who were much more likely to leave home in the subsequent ten years than younger children. Using a panel dataset constructed by hand-linking 1,600 male-headed households between the 1850 and 1860 censuses, Steckel (1996) calculated the number of children aged 5-6, 7-8, 9-10, etc. in 1850 who were still living in their parents' homes in 1860. After adjustment for suspected mortality, Steckel estimated that about 10.7% of boys aged 5-6 who were present in the 1850 census had left home before the 1860 census (when they would have been aged 15-16), while 11.5% of girls did so. Among children aged 9-10, the percentages increased to 22.1 and 43.1 respectively. The associated singulate mean ages at departure were 25.2 for males and 24.4 for females. Rates of leaving home were higher on the western frontier than in the Northeast, North-central, and South.

We suspect that the percentages of male and female children aged 5-6 in Census A who left home before Census B are lower in our panel datasets than Steckel's estimates for several reasons. First, Steckel assumed children in the 1850s experienced age-specific

mortality rates equivalent to Model West life table level 10. Under those conditions, 4.96% of male children exact age 5 in 1850 could expect to die before reaching exact age 15 in 1860. Other life table estimates for the period, including more recent estimates made by Haines (1998) and Hacker (2010), suggest higher mortality rates. The Meech life table for the period 1830-1860 (Meech 1898) suggests that 6.76% of male children exact age 5 died before age 15; the Jacobson 1850 life table indicates 10.3% (Jacobson 1957); Haines' 1850 life table indicates 5.15% (Haines 1998); and Hacker's life table for the 1850s 6.41% (Hacker 2010). If the actual rate of mortality experienced by boys in Steckel's sample was higher than the 4.96% rate he assumed, the true percentages of children who left home before the 1860 census was lower than he estimated.

Second, Steckel's dataset was based on a random sample of male household heads in 1860 with one or more children above the age of 10. The sampling strategy did not consider whether the male household head had a spouse present in the household in 1860, whether there was a spouse present in the 1850 census, or whether the spouse present in 1860 was the same spouse present in 1850. Children whose mothers died in the intercensal period likely experienced higher rates of leaving home than children whose mothers survived. Our panel datasets, in contrast, are limited to men and women who remained married to the same partner in both censuses. As a result, we suspect that the percentage of children in their mid-teenage years who had left home was lower in our panel dataset than in Steckel's dataset, and consequently, that a much higher percentage of children who were not present in Census B in our dataset were deceased rather than alive and living elsewhere.

Nonetheless, if there were significant differences in the age children left home by parents' wealth, those differences may bias our mortality analysis. Steckel's research, and that of a few other social scientists (e.g., Stevens 1990, who analyzes 1900 census data and

Gutmann *et al.* 2002, who studied the age at leaving home in the twentieth century), suggests that children of wealthier parents, who enjoyed more education and opportunities to work in a family business, may have been less likely to leave home at an earlier age than children of poorer parents. Unfortunately, the association between parents' wealth or class and the age at leaving home are difficult to estimate with census data.

To assess the potential for unobserved departure from children's parental home to bias our results, we estimated child mortality rates using our method by single years of age and compared the results to estimates from a model life table (Model West levels 6, 8, and 10, which we assumed encompasses the probable range of child mortality at the national level experienced during the period). When estimating the rates, we assumed that undercounted children in Census A died at the same rates as enumerated children. The results are shown in Figure A2. Our estimated results are lower at age 0 and typically higher for children at older ages, with a growing divergence at ages above 3.

The increasing departure of our estimates from those suggested by the model life tables at older ages are very likely the result of children leaving home. The age-specific shape of our results suggest unobserved child departures may have been present from a very early age. The divergence was not significant, however, until age 4 (corresponding to children aged 14 in Census B), when the estimated mortality of children in all three panel datasets was higher than the rate suggested by Model West level 6. By limiting our analysis to children aged 1-3 in Census A, we ensured that our estimates were dominated by child mortality, rather than the unobserved departure of children from home, and were not a risk from significant differentials in undercounting that may have been significant among children aged 0.

We constructed several alternate regression models to determine the robustness of our results for different age groups of children. We also tested models with the alternative child mortality estimate discussed above that assumed all unmatched children in Census B were undercounted and all unmatched children in Census A were deceased. Finally, we tested the results using an alternative age-adjusted measure of couples' wealth, where couples' wealth decile is determined for each age group of spouses. All results are shown in Figure A3, with our preferred estimate shown in black.

With only a few exceptions, the results were remarkably robust to different measures and age groups, including age groups that used infants in Census A. The mortality gradient when using age-adjusted wealth deciles was marginally less steep than when using unadjusted wealth (with mother's age and spouse's age differential included in the model), but very similar. Differences between child mortality measures using different age groups in Census A were modest for grouping in which younger children dominate (0-3, 0-4, 1-3, 1-4), as were differences when using the "high" mortality estimate discussed above (labeled alternative estimate in the figure). The only significant departure occurred with older aged children (children aged 2-6 in Census A). In these cases, the gradient was much steeper. This result is no doubt spurious, caused by the unobserved departure of greater proportions of children from homes with poorer parents than wealthier parents. This result suggests we should be cautious and interpreting our results and future work should explore the living arrangements of children with two surviving parents in more detail. Overall, however, the results were remarkably robust to different configurations of younger children, increasing the confidence of our finding of a negative gradient between wealth and child mortality.

Reliability of real and personal estate wealth data

A few nineteenth-century census officials and subsequent historians have expressed skepticism about the reliability of wealth data collected by the 1850, 1860, and 1870 censuses. Before conducting our fertility analysis, we examined the wealth data collected by each census. Two types of error were readily apparent. There was clear evidence of rounding at some values (especially at multiples of $100, $250, $500, and $1000). Second, a substantial number of married couples reported zero personal estate wealth in 1860 (12.6%) and 1870 (23.4%), both of which seemed unlikely. To some extent, the differences between 1860 and 1870 may be explained by the loss of wealth during the Civil War (1861-1865) and instructions to 1870 census enumerators to not report wealth under $100. On aggregate, however, the results appear to be good. As shown in Figure A4, mean wealth rose steadily with men's age until about age 65, after which it declined modestly. As noted in the main text, this age pattern was expected; men's age-related debilities increased with age, work hours and incomes declined, and wealth bequests were increasingly made to children reaching adulthood (Kearl and Pope 1983). And as expected, the regional patterns of wealth shifted after the war.

Table A1 shows OLS regression results for men's total wealth in 1860. Independent variables included the IPUMS "occscore" variable—an estimate in hundreds of dollars for the annual income in 1950 associated with each male's occupation—age, region, urban-rural residence, and nativity (U.S. born or foreign born). All results are as expected. Wealth was higher in South, in urban areas, increased with age, and higher among native-born men than foreign-born men. Most importantly, even though occupational income scores were taken from occupational earnings nearly a century later, wealth and occupational income score

were strongly and positively correlated, increasing confidence in the nineteenth-century wealth data. We conclude that that while wealth might be inconsistently recorded for some individuals, on aggregate the results appear to be excellent.

Complete Regression Results

In Tables A2, A3, and A4, we provide the complete regression results used to generate Figures 1-3 in the main text. In all census years, the results indicated higher mortality among children born to older mothers, among children whose parents were living in urban areas, and among children who had more siblings of a similar age. All else being equal, parents' literacy was only modestly and inconstantly correlated with lower child mortality, suggesting that greater parental knowledge was not a significant factor in child mortality differentials. Somewhat surprisingly, in most models we found lower mortality among children of parents born in Germany, Great Britain, Canada, and other foreign countries relative to children of parents born in the United States. In a few models, we even found lower mortality among children of Irish parents. In 1870, for example, the results indicated significantly lower mortality among children born to parents from Great Britain, Canada, and Germany. Most of these differences, however, only emerged in models controlling for urban-rural residence, region, wealth, and number of children exposed to the risk of mortality. Overall, children in the 1870 census born to foreign-born parents were more likely to live in the Northeast census region, in urban areas, and were born to parents with less wealth than children of native-born parents, which increased their overall risks (see Table 2). By the early twentieth century, children of native-born parents enjoyed significantly

lower mortality than children of most groups of foreign-born parents (Preston *et al.* 1994; Dribe *et al.* 2020).

Alternate Regression Results using Inverse Propensity Scores

As a final sensitivity test, we repeated the complete regression results for the 1860-1870 and 1870-1880 panel datasets using inverse propensity-score weights as described by Bailey, Cole, and Massey (2020). Couples under-represented in the panel datasets relative to the cross-sectional population in 1870 received higher weights, while over-represented couples received lower weights. We determined the propensity weights by regressing whether couples in the IPUMS complete-count 1870 dataset were linked to the subsequent census using the same selection criteria used in the models. The logistic regression model included covariates for census region, total wealth decile, literacy, occupation group, and number of children present in the household.

The results of models with and without propensity weights are shown in Table A5. Differences in most coefficients are very small. Coefficients indicating the wealth-fertility relationship were almost identical. In short, the IPUMS MLP datasets did not appear to have significant selection biases—as least as it applied to the population of married couples of childbearing ages in the period between 1850 and 1880—and weighting the results by propensity scores established using the cross-sectional IPUMS complete-count datasets had a negligible impact on the results.

References

Bailey, Martha, Cole, Connor, and Massey, Catherine G. (2020) "Simple strategies for improving inference with linked data: A case study of the 1850-1930 IPUMS Linked representative historical samples." Historical Methods: A Journal of Quantitative and Interdisciplinary History **53** (2): 80-93.

Dribe, Martin, J. David Hacker, and Francesco Scalone (2020) "Immigration and child mortality: Lessons from the United States at the turn of the twentieth century." Social Science History **44** (1): 57–89.

Gutmann, Myron P., Sara M. Pullum-Piñón, and Thomas W. Pullum (2002) "Three eras of young adult home leaving in twentieth-century America." Journal of Social History **35** (3): 533–76.

Hacker, J. David (2010) "Decennial life tables for the White population of the United States, 1790–1900." Historical Methods: A Journal of Quantitative and Interdisciplinary History **43** (2): 45–79.

________. (2013) "New estimates of census coverage in the United States, 1850-1930." Social Science History (37): 71–101.

Haines, Michael R. (1998) "Estimated life tables for the United States, 1850–1910." Historical Methods: A Journal of Quantitative and Interdisciplinary History **31** (4): 149–69.

Helgertz, Jonas, Joseph Price, Jacob Wellington, Kelly J. Thompson, Steven Ruggles, Catherine A. Fitch (2022) "A new strategy for linking U.S. historical censuses: A case study for the IPUMS multigenerational longitudinal panel." Historical Methods: A Journal of Quantitative and Interdisciplinary History **55** (1): 11-28.

Jacobson, Paul H. (1957) "An estimate of the expectation of life in the United States in 1850." Milbank Memorial Fund Quarterly **35** (2): 197–201.

Kearl, J. R. and Clayne L. Pope (1983) "The life cycle in economic history." Journal of Economic History **43** (1): 149–158.

Meech, Levi W. (1898) System and Tables of Life Insurance. Spectator.

Steckel, Richard H. (1996) "The age at leaving home in the United States, 1850-1860." Social Science History **20** (4): 507–532.

Preston, Samuel H., Ewbank, Douglas, and Mark Hereward (1994) "Child mortality differences by ethnicity and race in the United States : 1900-1910," in Susan Cotts Watkins (ed.) After Ellis Island. Newcomers and Natives in the 1910 Census. New York, Russell Sage Foundation: 35-82.

Stevens, David A. (1990). "New evidence on the timing of early life course transitions: The United States, 1900 to 1980." Journal of Family History **15** (1): 163–78.

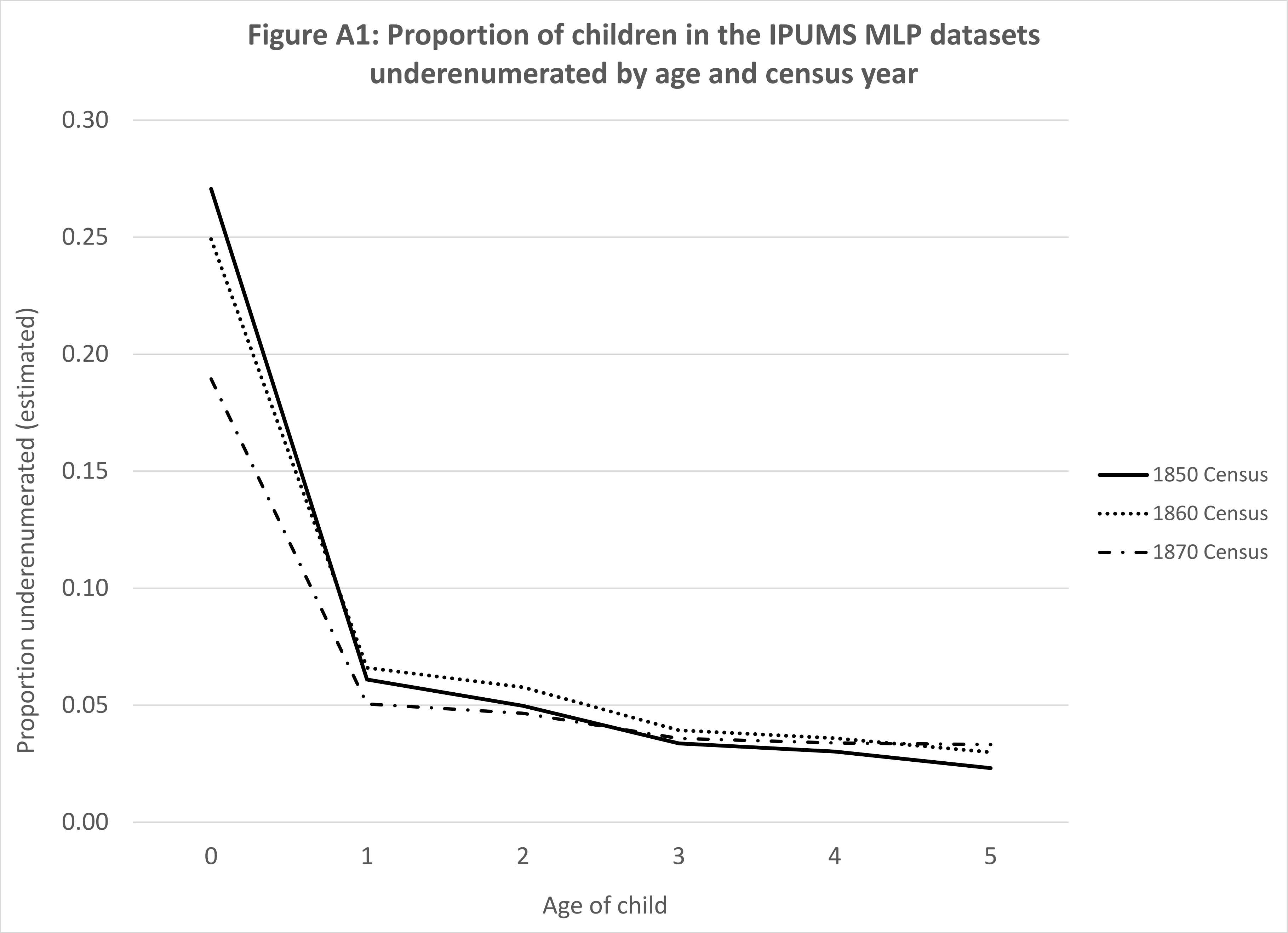
Figure A1: Proportion of children in the IPUMS MLP datasets underenumerated by age and census year
0.30
0.25
0.20
0.15
0.10
0.05
0.00
Proportion underenumerated (estimated)
0
1
2
3
4
5
Age of child
1850 Census
1860 Census
1870 Census

Figure A2: Comparison of estimated proportion of children age *x* dying before age *x*+10, intercensal interval in IPUMS MLP datasets and in selected model West life tables

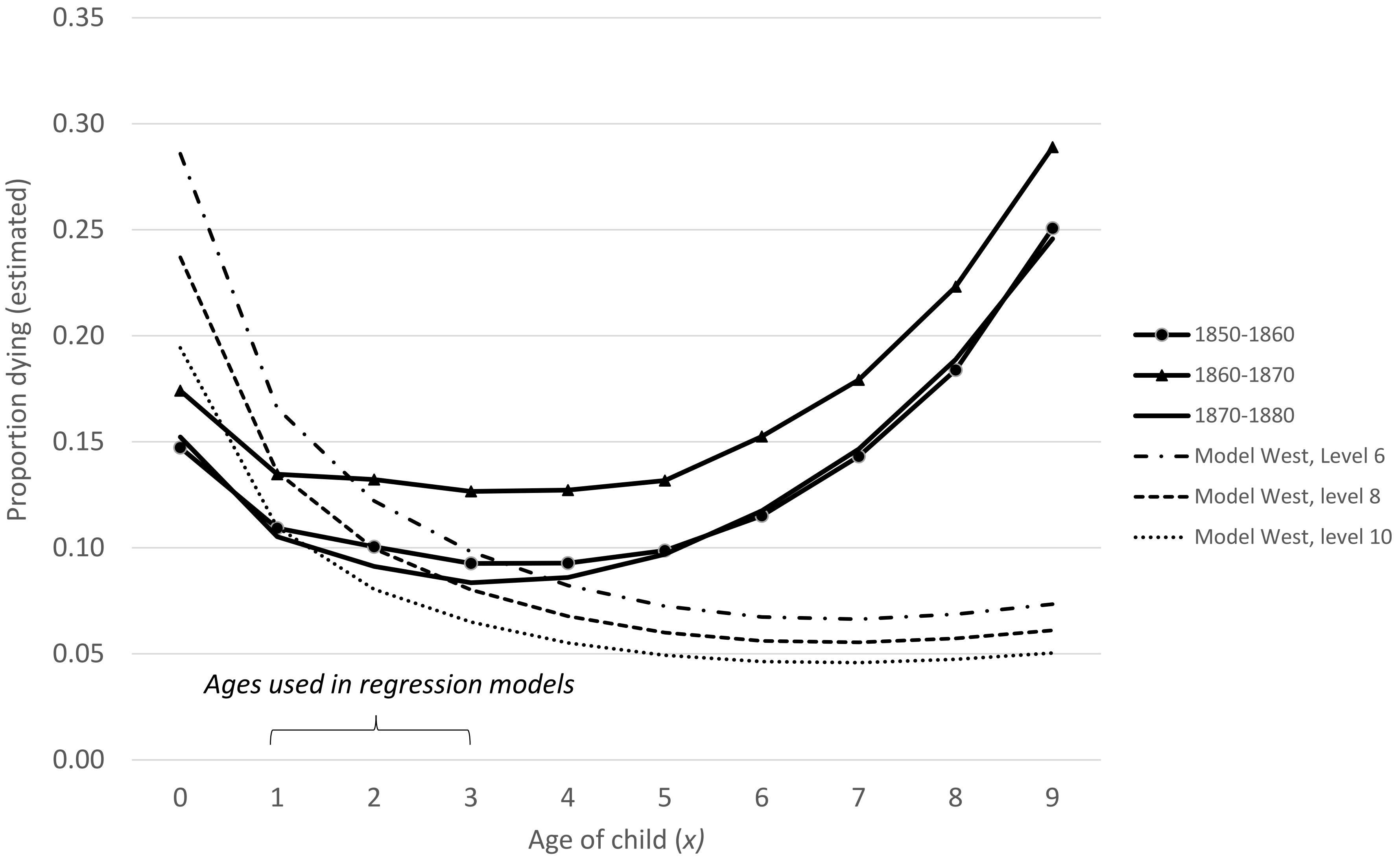

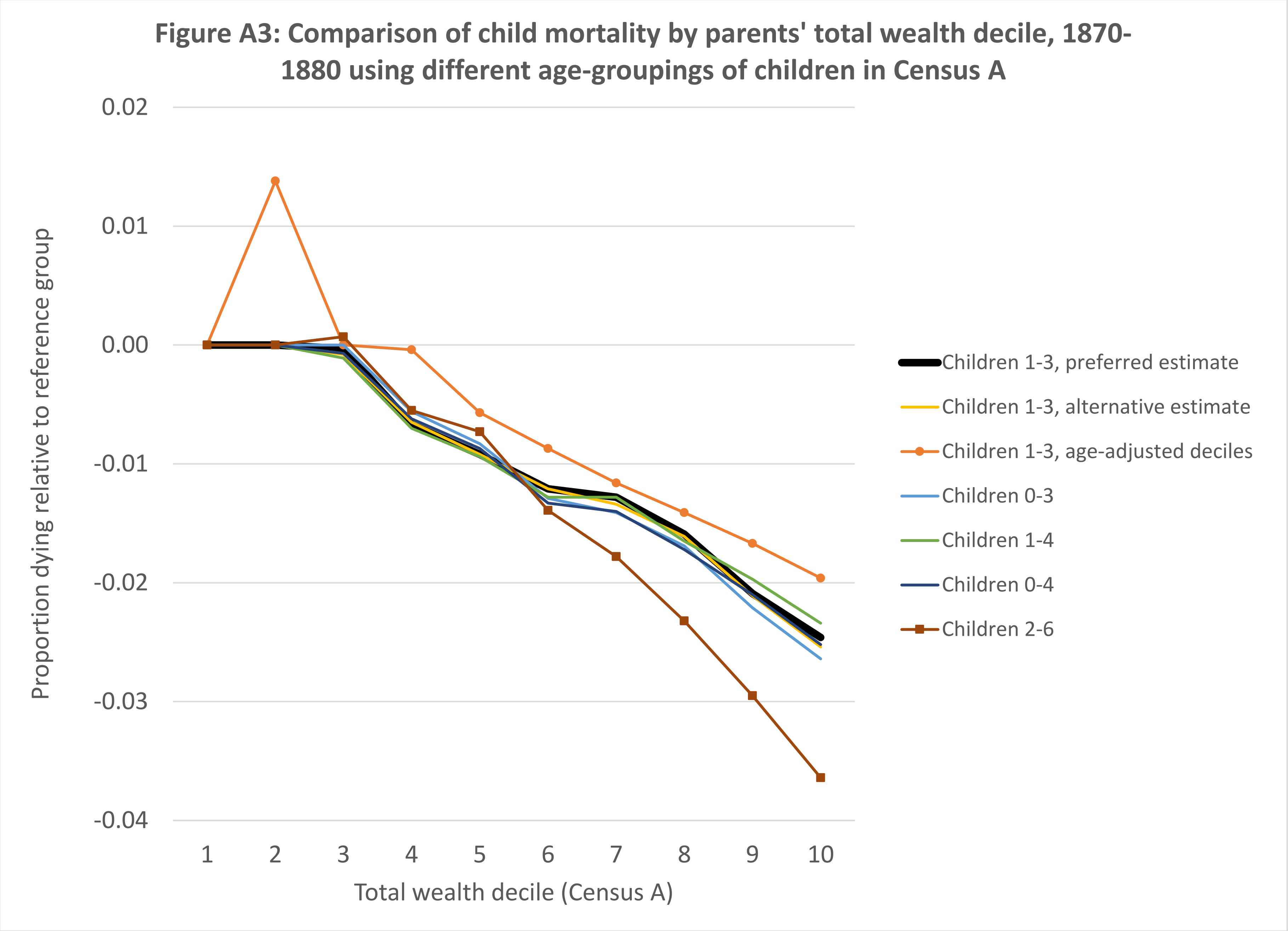

Figure A3: Comparison of child mortality by parents' total wealth decile, 1870-1880 using different age-groupings of children in Census A
0.02
0.01
0.00
-0.01
-0.02
-0.03
-0.04
Proportion dying relative to reference group
1
2
3
4
5
6
7
8
9
10
Total wealth decile (Census A)
Children 1-3, preferred estimate
Children 1-3, alternative estimate
Children 1-3, age-adjusted deciles
Children 0-3
Children 1-4
Children 0-4
Children 2-6

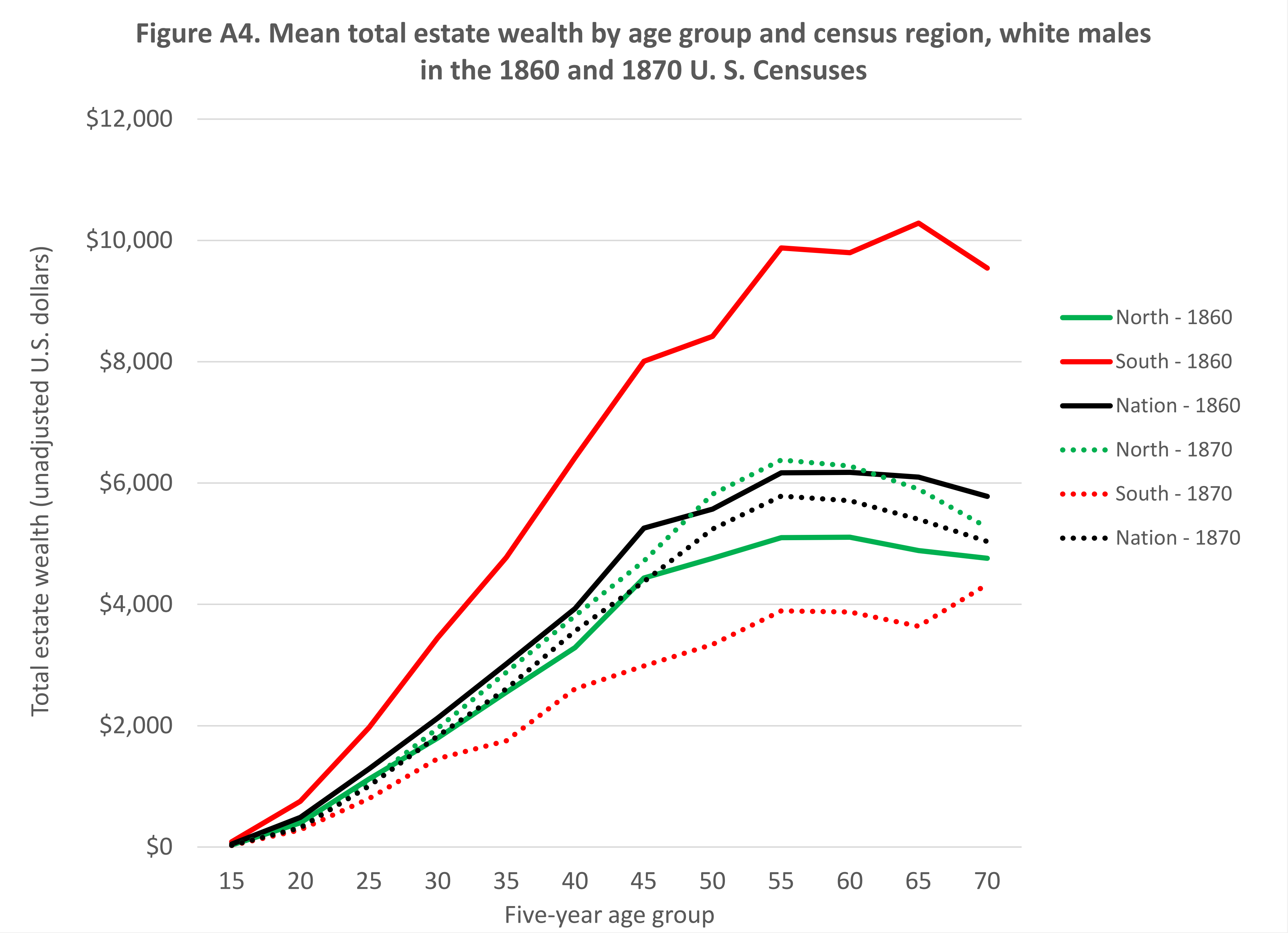
Figure A4. Mean total estate wealth by age group and census region, white males in the 1860 and 1870 U. S. Censuses
$12,000
$10,000
$8,000
$6,000
$4,000
$2,000
$0
Total estate wealth (unadjusted U.S. dollars)
15
20
25
30
35
40
45
50
55
60
65
70
Five-year age group
North - 1860
South - 1860
Nation - 1860
North - 1870
South - 1870
Nation - 1870

Table A1. OLS regression, total wealth of white males in 1860 census

Occupational income score	57.3	***
Age group		
15-19	-3611.6	***
20-24	-3397.4	***
25-29	-2606.6	***
30-34	-1740.3	***
35-39	-913.9	***
40-44	*ref.*	
45-49	1228.2	***
50-54	1598.1	***
55-59	2162.3	***
60-64	2269.0	***
65-69	2157.5	***
70-74	1946.9	***
Nativity		
U.S. born	*ref.*	
Foreign Born	-1737.2	***
Urban-Rural Residence		
Rural	*ref.*	
Urban	177.4	*
Census Region		
Northeast	*ref.*	
Midwest	189.5	**
South	1576.5	***
West	-464.7	**
Constant	2956.2	***

Source: 10% sample IPUMS 1860 complete dataset (Ruggles et al. 2020)

Table A2. Complete OLS Regression Results of Child Mortality in Intercensal Interval. Models with Parents' Real Estate Wealth.

	Linked Couples in the 1850-1860 IPUMS MLP Dataset					
	Nation	Northeast	Midwest	South	Non-Agricultural, urban	Agricultural occupations, rural
Mother's age group						
20-24 (ref.)	0.000	0.000	0.000	0.000	0.000	0.000
25-29	0.006 ***	0.008 ***	0.004	0.006 **	0.013 **	0.004 **
30-34	0.013 ***	0.017 ***	0.009 ***	0.014 ***	0.020 ***	0.012 ***
35-39	0.022 ***	0.027 ***	0.018 ***	0.018 ***	0.031 ***	0.018 ***
40-44	0.030 ***	0.032 ***	0.031 ***	0.029 ***	0.026 **	0.031 ***
45-49	0.032 *	0.027	0.032	0.041	-0.011	0.039 *
Age difference spouse	0.001 ***	0.001 ***	0.001 ***	0.000 **	0.001	0.001 ***
Parents' Nativity						
United States (ref.)	0.000	0.000	0.000	0.000	0.000	0.000
Germany	-0.007 **	-0.010 *	-0.010 **	0.001	-0.005	-0.013 **
Ireland	-0.013 ***	-0.012 ***	-0.020 ***	-0.026 **	-0.015 ***	-0.019 ***
Great Britain	-0.007 **	-0.009 **	-0.008 *	0.000	-0.004	-0.008 *
Canada	-0.002	-0.001	-0.007	0.056	0.016	-0.004
Other Foreign	0.001	-0.014	0.009	0.013	-0.022 *	0.007
Literate	0.000	-0.012 ***	0.007 ***	-0.002	-0.022 **	0.003
Residence type						
Rural (ref.)	0.000	0.000	0.000	0.000		
Urban, less than 10,000 pop.	0.016 ***	0.007	0.032 ***	0.017 *	0.000	
Urban, 10,000-24,999	0.018 ***	0.018 ***	0.024 **	-0.008	0.008	
Urban, 25,000-99,999	0.024 ***	0.019 **	0.039	0.025 **	0.006	
Urban, 100,000 plus	0.015	0.001	0.048 ***	0.006 ***	0.003	
Number of children aged 1-3 at risk	0.010 ***	0.011 ***	0.010 ***	0.009 ***	0.007 *	0.010 ***
Parents' Real Estate Wealth						
Decile 1 - no wealth (ref.)	0.000	0.000	0.000	0.000	0.000	0.000
Decile 2 - no wealth (ref.)	0.000	0.000	0.000	0.000	0.000	0.000
Decile 3 - no wealth (ref.)	0.000	0.000	0.000	0.000	0.000	0.000
Decile 4 - no wealth (ref.)	0.000	0.000	0.000	0.000	0.000	0.000
Decile 5	-0.004 **	-0.004	0.000	-0.006 **	0.001	-0.001
Decile 6	-0.007 ***	-0.007 **	-0.004	-0.008 **	-0.005	-0.005 **
Decile 7	-0.010 ***	-0.012 ***	-0.006 *	-0.009 ***	-0.016 *	-0.006 ***
Decile 8	-0.011 ***	-0.017 ***	-0.003	-0.010 **	0.003	-0.008 ***
Decile 9	-0.012 ***	-0.017 ***	-0.011 ***	-0.004	0.006	-0.007 **
Decile 10	-0.015 ***	-0.025 ***	-0.007 *	-0.004	-0.014 *	-0.005 **
Number of couples	539,235	215,586	164,864	157,795	46,066	311,450
r-square	0.011	0.009	0.010	0.014	0.007	0.005

	Linked Couples in the 1860-1870 IPUMS MLP Dataset					
	Nation	Northeast	Midwest	South	Non-Agricultural, urban	Agricultural occupations, rural
Mother's age group						
20-24 (ref.)						
25-29	0.001	0.006 *	0.001	-0.003	0.005	0.001
30-34	0.005 ***	0.007 ***	0.005 **	0.004	0.006	0.006 ***
35-39	0.010 ***	0.013 ***	0.010 ***	0.007 **	0.008	0.013 ***
40-44	0.017 ***	0.020 ***	0.015 **	0.014 *	0.008	0.024 ***
45-49	0.031 *	0.089 **	0.010	-0.010	0.016	0.021
Age difference spouse	0.000 **	0.000	0.000 *	0.000	0.000	0.000 *
Parents' Nativity						
United States (ref.)	0.000	0.000	0.000	0.000	0.000	0.000
Germany	-0.006 **	-0.008 *	-0.006 **	-0.006	-0.005	-0.002
Ireland	-0.007 **	-0.006	-0.017 ***	0.005	-0.001	-0.014 ***
Great Britain	-0.010 ***	-0.011 ***	-0.009 ***	-0.015 *	-0.014 **	-0.009 **
Canada	-0.008 **	-0.011 **	-0.006	-0.038	-0.014 **	-0.004

Other Foreign	-0.017 ***	-0.024 ***	-0.017 ***	-0.018 *	-0.023 ***	-0.014 ***
Literate	-0.001	-0.007 *	0.000	0.002	-0.009	-0.001
Residence type						
Rural (ref.)	0.000	0.000	0.000	0.000		
Urban, less than 10,000 pop.	0.005	0.003	0.006	0.011	0.000	
Urban, 10,000-24,999	0.010 *	0.005	0.020 **	0.018	0.001	
Urban, 25,000-99,999	0.011 *	0.012 *	0.002	0.012 ***	0.003	
Urban, 100,000 plus	0.000	0.004	0.002	-0.049 ***	-0.003	
Number of children aged 1-3 at risk	0.008 ***	0.009 ***	0.008 ***	0.003 *	0.006 **	0.008 ***
Parents' Real Estate Wealth						
Decile 1 - no wealth (ref.)	0.000	0.000	0.000	0.000	0.000	0.000
Decile 2 - no wealth (ref.)	0.000	0.000	0.000	0.000	0.000	0.000
Decile 3 - no wealth (ref.)	0.000	0.000	0.000	0.000	0.000	0.000
Decile 4 - no wealth (ref.)	0.000	0.000	0.000	0.000	0.000	0.000
Decile 5	-0.002	0.000	0.001	-0.005 *	0.000	-0.001
Decile 6	-0.008 ***	-0.008 **	-0.006 **	-0.009 ***	-0.007 *	-0.006 ***
Decile 7	-0.012 ***	-0.012 ***	-0.011 ***	-0.012 ***	-0.009 *	-0.010 ***
Decile 8	-0.012 ***	-0.012 ***	-0.011 ***	-0.013 ***	-0.007	-0.009 ***
Decile 9	-0.014 ***	-0.016 ***	-0.012 ***	-0.013 ***	-0.009	-0.011 ***
Decile 10	-0.015 ***	-0.025 ***	-0.009 ***	-0.008 *	-0.014 ***	-0.011 ***
Number of couples	750,469	277,673	297,968	164,668	115,648	358,896
r-square	0.009	0.006	0.006	0.016	0.007	0.005

	Linked Couples in the 1860-1870 IPUMS MPL Dataset					
	Nation	Northeast	Midwest	South	Non-Agricultural, urban	Agricultural occupations, rural
Mother's age group						
20-24 (ref.)	0.000	0.000	0.000	0.000	0.000	0.000
25-29	0.002	0.001	0.003 *	0.000	0.002	0.002
30-34	0.007 ***	0.008 ***	0.006 ***	0.006 ***	0.003	0.007 ***
35-39	0.013 ***	0.010 ***	0.015 ***	0.014 ***	0.009 **	0.014 ***
40-44	0.023 ***	0.022 ***	0.025 ***	0.023 ***	0.020 **	0.024 ***
45-49	0.012	-0.001	0.008	0.049 *	-0.023	0.019
Age difference spouse	0.000 ***	0.000 *	0.000 ***	0.000 *	0.000	0.001 ***
Parents' Nativity						
United States (ref.)	0.000	0.000	0.000	0.000	0.000	0.000
Germany	0.003	0.005	0.001	0.006	0.006 *	0.001
Ireland	-0.005 *	-0.002	-0.012 ***	0.001	0.001	-0.008 ***
Great Britain	-0.006 ***	-0.002	-0.010 ***	-0.001	-0.003	-0.009 ***
Canada	0.003	0.005	0.000	-0.010	-0.001	-0.001
Other Foreign	-0.008 ***	-0.012	-0.008 ***	-0.014 *	-0.011 **	-0.004
Literate	-0.005 ***	-0.009 ***	-0.003 *	-0.003	-0.011 ***	-0.002 *
Residence type						
Rural (ref.)	0.000	0.000	0.000	0.000		
Urban, less than 10,000 pop.	0.007 ***	0.007	0.007 ***	0.002	0.000	
Urban, 10,000-24,999	0.011 ***	0.014 ***	0.009 *	-0.007	0.005	
Urban, 25,000-99,999	0.015 ***	0.014 ***	0.021 ***	-0.001	0.010	
Urban, 100,000 plus	0.021 ***	0.023 ***	0.020 *	0.022 ***	0.010	
Number of children aged 1-3 at risk	0.014 ***	0.012 ***	0.015 ***	0.014 ***	0.014 ***	0.014 ***
Parents' Real Estate Wealth						
Decile 1 - no wealth (ref.)	0.000	0.000	0.000	0.000	0.000	0.000
Decile 2 - no wealth (ref.)	0.000	0.000	0.000	0.000	0.000	0.000
Decile 3 - no wealth (ref.)	0.000	0.000	0.000	0.000	0.000	0.000
Decile 4 - no wealth (ref.)	0.000	0.000	0.000	0.000	0.000	0.000
Decile 5	-0.001	0.002	0.001	-0.005 *	-0.010	-0.002
Decile 6	-0.005 ***	-0.002	-0.004 *	-0.011 ***	-0.006 *	-0.007 ***
Decile 7	-0.007 ***	-0.004 *	-0.009 ***	-0.007 **	-0.003	-0.007 ***
Decile 8	-0.011 ***	-0.010 ***	-0.012 ***	-0.010 ***	-0.007 *	-0.010 ***
Decile 9	-0.015 ***	-0.014 ***	-0.017 ***	-0.010 ***	-0.017 ***	-0.014 ***
Decile 10	-0.020 ***	-0.024 ***	-0.019 ***	-0.013 ***	-0.020 ***	-0.017 ***

Number of couples	1,027,252	341,499	443,895	218,777	201,161	538,600
r-square	0.011	0.008	0.008	0.017	0.008	0.007

Notes: The dependent variable is the proportion of children age 1-3 in Census A dying prior to Census B. Results are weighted by the number of children at risk of death. The models for the nation and each of the three major regions employ county-level fixed effects. The models for the non-agricultural, urban and the agricultural, rural populations employ SEA-level fixed effects. In the model for the urban, non-agricultural population, the reference group for residence type is urban, less than 10,000 population.

Table A3. Complete OLS Regression Results of Child Mortality in Intercensal Interval. Models with Parents' Personal Estate Wealth

	Linked Couples in the 1860-1870 IPUMS MLP Dataset					
	Nation	Northeast	Midwest	South	Non-Agricultural, urban	Agricultural occupations, rural
Mother's age group						
20-24 (ref.)	0.000	0.000	0.000	0.000	0.000	0.000
25-29	0.001	0.006 *	0.000	-0.004	0.005	0.001
30-34	0.005 ***	0.007 ***	0.004 *	0.004	0.006	0.006 ***
35-39	0.010 ***	0.013 ***	0.009 ***	0.007 **	0.008	0.013 ***
40-44	0.016 ***	0.020 ***	0.015 **	0.014 *	0.008	0.024 ***
45-49	0.031 *	0.088 **	0.010	-0.011	0.016	0.021
Age difference spouse	0.000 **	0.000	0.000 *	0.000	0.000	0.000 *
Parents' Nativity						
United States (ref.)	0.000	0.000	0.000	0.000	0.000	0.000
Germany	-0.007 ***	-0.009 *	-0.007 **	-0.008	-0.007 *	-0.004
Ireland	-0.010 ***	-0.009 **	-0.018 ***	0.002	-0.005	-0.016 ***
Great Britain	-0.011 ***	-0.012 ***	-0.009 ***	-0.016 *	-0.015 **	-0.009 **
Canada	-0.008 **	-0.012 **	-0.007	-0.037	-0.014 **	-0.004
Other Foreign	-0.018 ***	-0.025 ***	-0.018 ***	-0.018 *	-0.024 ***	-0.015 ***
Literate	0.000	-0.006 *	0.000	0.003	-0.007	0.000
Residence type						
Rural (ref.)	0.000	0.000	0.000	0.000		
Urban, less than 10,000 pop.	0.003	0.003	0.004	0.010	0.000	
Urban, 10,000-24,999	0.009 *	0.006	0.019 *	0.017	0.001	
Urban, 25,000-99,999	0.012 *	0.014 *	0.002	0.012 ***	0.003	
Urban, 100,000 plus	0.000	0.000	0.002	-0.049 ***	-0.001	
Number of children aged 1-3 at risk	0.008 ***	0.009 ***	0.008 ***	0.003 *	0.006 **	0.008
Parents' Personal Estate Wealth						
Decile 1 - no wealth (ref.)	0.000	0.000	0.000	0.000	0.000	0.000
Decile 2	0.006 **	0.004	0.007 *	0.008	0.007	0.002
Decile 3	0.001	0.002	0.001	-0.002	-0.004	0.000
Decile 4	-0.005 **	-0.004	-0.004	-0.008 *	-0.005	-0.004
Decile 5	-0.012 ***	-0.013 ***	-0.008 **	-0.016 ***	-0.015 ***	-0.011 ***
Decile 6	-0.011 ***	-0.009 **	-0.009 **	-0.013 **	0.000	-0.009 ***
Decile 7	-0.010 ***	-0.013 ***	-0.007 *	-0.010 *	-0.007	-0.010 ***
Decile 8	-0.015 ***	-0.017 ***	-0.013 ***	-0.015 ***	-0.016 *	-0.013 ***
Decile 9	-0.020 ***	-0.024 ***	-0.015 ***	-0.020 ***	-0.020 ***	-0.020 ***
Decile 10	-0.015 ***	-0.026 ***	-0.010 **	-0.012 **	-0.021 ***	-0.015 ***
Number of couples	750,469	277,673	297,968	164,668	115,648	358,896
r-square	0.009	0.006	0.006	0.016	0.008	0.006

	Linked Couples in the 1870-1880 IPUMS MLP Dataset					
	Nation	Northeast	Midwest	South	Non-Agricultural, urban	Agricultural occupations, rural
Mother's age group						
20-24 (ref.)	0.000	0.000	0.000	0.000	0.000	0.000
25-29	0.002	0.001	0.002	0.000	0.002	0.002
30-34	0.007 ***	0.008 ***	0.005 ***	0.007 ***	0.002	0.007 ***
35-39	0.012 ***	0.010 ***	0.013 ***	0.015 ***	0.008 *	0.014 ***
40-44	0.023 ***	0.022 ***	0.023 ***	0.024 ***	0.018 *	0.024 ***
45-49	0.011	-0.002	0.007	0.048 *	-0.025	0.019
Age difference spouse	0.000 ***	0.000 *	0.000 ***	0.000 *	0.000	0.000 ***
Parents' Nativity						
United States (ref.)	0.000	0.000	0.000	0.000	0.000	0.000
Germany	0.002	0.004	0.000	0.005	0.005 *	0.000
Ireland	-0.007 ***	-0.004	-0.014 ***	-0.001	-0.001	-0.009 ***
Great Britain	-0.006 ***	-0.003	-0.011 ***	-0.002	-0.003	-0.009 ***

Canada	0.002	0.004	0.000	-0.010	-0.002	-0.001
Other Foreign	-0.009 ***	-0.012	-0.008 ***	-0.014 *	-0.011 **	-0.004
Literate	-0.004 ***	-0.008 ***	-0.003 *	-0.002	-0.010 ***	-0.002
Residence type						
Rural (ref.)	0.000	0.000	0.000	0.000		
Urban, less than 10,000 pop.	0.006 **	0.006	0.006 **	0.001	0.000	
Urban, 10,000-24,999	0.010 ***	0.014 ***	0.007	-0.007	0.005	
Urban, 25,000-99,999	0.014 ***	0.014 ***	0.020 ***	-0.001	0.010	
Urban, 100,000 plus	0.021 ***	0.023 **	0.021 *	0.019 ***	0.010	
Number of children aged 1-3 at risk	0.014 ***	0.012 ***	0.015 ***	0.014 ***	0.014 ***	0.014 ***
Parents' Personal Estate Wealth						
Decile 1 - no wealth (ref.)	0.000	0.000	0.000	0.000	0.000	0.000
Decile 2 - no wealth (ref.)	0.000	0.000	0.000	0.000	0.000	0.000
Decile 3	-0.002	-0.003	-0.002	-0.002	-0.004	-0.002
Decile 4	-0.004 ***	-0.004	-0.003	-0.008 **	-0.003	-0.003
Decile 5	-0.010 ***	-0.004 *	-0.010 ***	-0.016 ***	-0.008 **	-0.010 ***
Decile 6	-0.010 ***	-0.009 **	-0.010 ***	-0.013 ***	-0.011 **	-0.009 ***
Decile 7	-0.013 ***	-0.013 ***	-0.012 ***	-0.014 ***	-0.011 ***	-0.011 ***
Decile 8	-0.017 ***	-0.017 ***	-0.018 ***	-0.018 ***	-0.013 ***	-0.016 ***
Decile 9	-0.019 ***	-0.022 ***	-0.018 ***	-0.013 ***	-0.002	-0.018 ***
Decile 10	-0.023 ***	-0.029 ***	-0.021 ***	-0.017 ***	-0.026 ***	-0.021 ***
Number of couples	1,027,252	341,499	443,895	218,777	201,161	538,600
r-square	0.011	0.009	0.008	0.017	0.008	0.007

Notes: See Table A2.

Table A4. Complete OLS Regression Results of Child Mortality in Intercensal Interval. Models with Parents' Total Estate Wealth

	Linked Couples in the 1860-1870 IPUMS MLP Dataset					
	Nation	Northeast	Midwest	South	Non-Agricultural, urban	Agricultural occupations, rural
Mother's age group						
20-24 (ref.)	0.000	0.000	0.000	0.000	0.000	0.000
25-29	0.002	0.006 **	0.001	-0.003	0.005	0.001
30-34	0.006 ***	0.008 ***	0.005 **	0.004	0.007 *	0.006 ***
35-39	0.011 ***	0.014 ***	0.010 ***	0.007 **	0.009 *	0.013 ***
40-44	0.017 ***	0.021 ***	0.016 ***	0.015 *	0.009	0.024 ***
45-49	0.032 *	0.090 ***	0.011	-0.010	0.017	0.022
Age difference spouse	0.000 ***	0.000	0.000 **	0.000	0.000	0.000 **
Parents' Nativity						
United States (ref.)	0.000	0.000	0.000	0.000	0.000	0.000
Germany	-0.006 **	-0.009 *	-0.006 **	-0.007	-0.006	-0.003
Ireland	-0.009 ***	-0.008 **	-0.017 ***	0.003	-0.004	-0.015 ***
Great Britain	-0.011 ***	-0.013 ***	-0.009 ***	-0.016 *	-0.015 **	-0.009 **
Canada	-0.008 **	-0.012 **	-0.006	-0.038	-0.014 **	-0.004
Other Foreign	-0.018 ***	-0.024 ***	-0.017 ***	-0.018 *	-0.023 ***	-0.015 ***
Literate	0.000	-0.005	0.001	0.003	-0.007	0.000
Residence type						
Rural (ref.)	0.000	0.000	0.000	0.000		
Urban, less than 10,000 pop.	0.004	0.002	0.005	0.011	0.000	
Urban, 10,000-24,999	0.009 *	0.005	0.019 *	0.017	0.001	
Urban, 25,000-99,999	0.011 *	0.012 *	0.002	0.012 ***	0.002	
Urban, 100,000 plus	-0.001	-0.001	0.001	-0.050 ***	-0.003	
Number of children aged 1-3 at risk	0.008 ***	0.009 ***	0.008 ***	0.003 *	0.006 **	0.008 ***
Parents' Total Estate Wealth						
Decile 1 - no wealth (ref.)	0.000	0.000	0.000	0.000	0.000	0.000
Decile 2	0.005 **	0.005	0.008 *	0.002	0.002	0.005
Decile 3	-0.002	-0.002	0.003	-0.011 *	-0.008	0.000
Decile 4	-0.008 ***	-0.010 ***	-0.003	-0.013 ***	-0.010 **	-0.007 **
Decile 5	-0.011 ***	-0.009 **	-0.008 *	-0.014 ***	-0.013 **	-0.011 ***
Decile 6	-0.013 ***	-0.016 ***	-0.009 ***	-0.012 **	-0.014 ***	-0.010 ***
Decile 7	-0.015 ***	-0.017 ***	-0.010 ***	-0.020 ***	-0.014 *	-0.013 ***
Decile 8	-0.016 ***	-0.017 ***	-0.012 ***	-0.020 ***	-0.014 *	-0.015 ***
Decile 9	-0.017 ***	-0.021 ***	-0.011 ***	-0.021 ***	-0.019 ***	-0.015 ***
Decile 10	-0.018 ***	-0.032 ***	-0.011 ***	-0.013 **	-0.018 ***	-0.017 ***
Number of couples	750,469	277,673	297,968	164,668	115,648	358,896
r-square	0.010	0.006	0.006	0.016	0.007	0.006

	Linked Couples in the 1870-1880 IPUMS MLP Dataset					
	Nation	Northeast	Midwest	South	Non-Agricultural, urban	Agricultural occupations, rural
Mother's age group						
20-24 (ref.)	0.000	0.000	0.000	0.000	0.000	0.000
25-29	0.002 *	0.002	0.003 *	0.001	0.002	0.002
30-34	0.008 ***	0.009 ***	0.007 ***	0.007 ***	0.004	0.008 ***
35-39	0.014 ***	0.011 ***	0.015 ***	0.015 ***	0.010 **	0.015 ***
40-44	0.024 ***	0.024 ***	0.025 ***	0.024 ***	0.021 **	0.025 ***
45-49	0.012	0.000	0.008	0.049 *	-0.022	0.020
Age difference spouse	0.000 ***	0.000 **	0.000 ***	0.000 *	0.000	0.001 ***
Parents' Nativity						
United States (ref.)	0.000	0.000	0.000	0.000	0.000	0.000
Germany	0.003	0.005	0.001	0.006	0.006 *	0.001
Ireland	-0.006 ***	-0.003	-0.013 ***	0.000	-0.001	-0.009 ***
Great Britain	-0.006 ***	-0.003	-0.011 ***	-0.002	-0.003	-0.009 ***

Canada	0.002		0.004		0.000		-0.010		-0.002		-0.001	
Other Foreign	-0.008	***	-0.012		-0.008	***	-0.014	*	-0.011	**	-0.004	
Literate	-0.004	***	-0.008	***	-0.002		-0.002		-0.010	***	-0.002	
Residence type												
Rural (ref.)	0.000		0.000		0.000		0.000					
Urban, less than 10,000 pop.	0.007	***	0.007		0.007	**	0.002		0.000			
Urban, 10,000-24,999	0.010	***	0.014	***	0.008	*	-0.007		0.005			
Urban, 25,000-99,999	0.014	***	0.013	***	0.021	***	-0.001		0.010			
Urban, 100,000 plus	0.021	***	0.022	***	0.020	*	0.020	***	0.009			
Number of children aged 1-3 at risk	0.014	***	0.012	***	0.015	***	0.014	***	0.014	***	0.014	***
Parents' Total Estate Wealth												
Decile 1 - no wealth (ref.)	0.000		0.000		0.000		0.000		0.000		0.000	
Decile 2 - no wealth (ref.)	0.000		0.000		0.000		0.000		0.000		0.000	
Decile 3	0.000		-0.001		0.003		-0.004		-0.001		-0.001	
Decile 4	-0.007	***	-0.006	**	-0.004	*	-0.013	***	-0.009	***	-0.006	***
Decile 5	-0.009	***	-0.009	***	-0.008	***	-0.013	***	-0.005		-0.009	***
Decile 6	-0.012	***	-0.010	***	-0.011	***	-0.017	***	-0.010	***	-0.013	***
Decile 7	-0.013	***	-0.011	***	-0.013	***	-0.015	***	-0.011	***	-0.013	***
Decile 8	-0.016	***	-0.015	***	-0.016	***	-0.016	***	-0.012	***	-0.015	***
Decile 9	-0.021	***	-0.023	***	-0.020	***	-0.018	***	-0.021	***	-0.020	***
Decile 10	-0.025	***	-0.028	***	-0.023	***	-0.019	***	-0.028	***	-0.022	***
Number of couples	1,027,252		341,499		443,895		218,777		201,161		538,600	
r-square	0.011		0.009		0.008		0.017		0.008		0.007	

Notes: See Table A2.

Table A5. OLS Regression, Child Mortality in Intercensal Interval with Parents' Total Wealth Decile, Comparison of Results with and Without Propensity Weights

	Linked Couples in the 1860-1870 IPUMS MLP Dataset		Linked Couples in the 1870-1880 IPUMS MLP Dataset	
Propensity Weighting	No	Yes	No	Yes
Mother's age group				
20-24 (ref.)	0.000	0.000	0.000	0.000
25-29	-0.018 ***	-0.018 ***	0.000	0.000
30-34	-0.019 ***	-0.017 ***	0.000	0.000
35-39	0.002	0.002	0.002 *	0.002
40-44	0.006 ***	0.006 ***	0.008 ***	0.007 ***
45-49	0.012 ***	0.011 ***	0.014 ***	0.014 ***
Age difference spouse	0.000 ***	0.000 *	0.000 ***	0.000 ***
Parents' Nativity				
United States (ref.)	0.000	0.000	0.000	0.000
Germany	-0.006 **	-0.006 **	0.002	0.002
Ireland	-0.009 ***	-0.008 **	-0.006 ***	-0.006 **
Great Britain	-0.011 ***	-0.013 ***	-0.007 ***	-0.007 ***
Canada	-0.008 ***	-0.010 ***	0.002	0.001
Other Foreign	-0.018 ***	-0.021 ***	-0.009 ***	-0.009 ***
Literate	0.001	0.001	-0.004 ***	-0.005 ***
Residence type				
Rural (ref.)	0.000	0.000	0.000	0.000
Urban, less than 10,000 pop.	0.004	0.004	0.007 ***	0.006 ***
Urban, 10,000-24,999	0.009 *	0.008	0.010 ***	0.010 ***
Urban, 25,000-99,999	0.011 *	0.010 *	0.014 ***	0.015 ***
Urban, 100,000 plus	-0.001	-0.005	0.021 ***	0.021 **
Number of children aged 1-3 at risk	0.006 ***	0.005 ***	0.013 ***	0.012 ***
Parents' Total Estate Wealth				
Decile 1 - no wealth (ref.)	0.000	0.000	0.000	0.000
Decile 2	0.005 *	0.005 **	0.000	0.000
Decile 3	-0.003	-0.004	-0.001	-0.001
Decile 4	-0.009 ***	-0.009 ***	-0.007 ***	-0.008 ***
Decile 5	-0.012 ***	-0.011 ***	-0.010 ***	-0.010 ***
Decile 6	-0.014 ***	-0.013 ***	-0.013 ***	-0.013 ***
Decile 7	-0.012 ***	-0.011 ***	-0.013 ***	-0.014 ***
Decile 8	-0.014 ***	-0.013 ***	-0.016 ***	-0.016 ***
Decile 9	-0.016 ***	-0.016 ***	-0.021 ***	-0.021 ***
Decile 10	-0.017 ***	-0.017 ***	-0.025 ***	-0.025 ***
Number of couples	740,013	740,013	1,013,715	1,013,715
r-square	0.010	0.010	0.011	0.011

Notes: See Table A2. Models with Propensity Weights were Weighted to Reflect the Cross-sectional Populations in Census A.
